# Natural evidence of coronaviral 2′-O-methyltransferase activity affecting viral pathogenesis via improved substrate RNA binding

**DOI:** 10.1038/s41392-024-01860-x

**Published:** 2024-05-29

**Authors:** Jikai Deng, Shimin Yang, Yingjian Li, Xue Tan, Jiejie Liu, Yanying Yu, Qiang Ding, Chengpeng Fan, Hongyun Wang, Xianyin Chen, Qianyun Liu, Xiao Guo, Feiyu Gong, Li Zhou, Yu Chen

**Affiliations:** 1 State Key Laboratory of Virology, RNA Institute, College of Life Sciences and Frontier Science Center for Immunology and Metabolism, Wuhan University, Wuhan, China; 2 School of Medicine, Tsinghua University, Beijing, China; 3 School of Basic Medical Sciences, Wuhan University, Wuhan, China; 4 Animal Bio-Safety Level III Laboratory/Institute for Vaccine Research, Wuhan University School of Medicine, Wuhan, China

**Keywords:** Microbiology, Inflammation

## Abstract

Previous studies through targeted mutagenesis of K-D-K-E motif have demonstrated that 2′-O-MTase activity is essential for efficient viral replication and immune evasion. However, the K-D-K-E catalytic motif of 2′-O-MTase is highly conserved across numerous viruses, including flaviviruses, vaccinia viruses, coronaviruses, and extends even to mammals. Here, we observed a stronger 2′-O-MTase activity in SARS-CoV-2 compared to SARS-CoV, despite the presence of a consistently active catalytic center. We further identified critical residues (Leu-36, Asn-138 and Ile-153) which served as determinants of discrepancy in 2′-O-MTase activity between SARS-CoV-2 and SARS-CoV. These residues significantly enhanced the RNA binding affinity of 2′-O-MTase and boosted its versatility toward RNA substrates. Of interest, a triple substitution (Leu^36^ → Ile^36^, Asn^138^ → His^138^, Ile^153^ → Leu^153^, from SARS-CoV-2 to SARS-CoV) within nsp16 resulted in a proportional reduction in viral 2′-O-methylation and impaired viral replication. Furthermore, it led to a significant upregulation of type I interferon (IFN-I) and proinflammatory cytokines both in vitro and *vivo*, relying on the cooperative sensing of melanoma differentiation-associated protein 5 (MDA5) and laboratory of genetics and physiology 2 (LGP2). In conclusion, our findings demonstrated that alterations in residues other than K-D-K-E of 2′-O-MTase may affect viral replication and subsequently influence pathogenesis. Monitoring changes in nsp16 residues is crucial as it may aid in identifying and assessing future alteration in viral pathogenicity resulting from natural mutations occurring in nsp16.

## Introduction

Coronaviruses (CoVs) are prevalent pathogens that can infect both humans and other mammal. Highly pathogenic coronavirus strains, such as SARS-CoV and SARS-CoV-2, are known to cause severe acute respiratory syndrome (SARS) and COVID-19, posing a serious challenge to human health.^1^ Both SARS-CoV-2 and SARS-CoV, members of the Betacoronavirus genus, are enveloped viruses characterized by a single-stranded, positive-sense RNA genome, which is about 30 kb in length.^2^ The genomes feature a 5′-cap structure at one end and a 3′-poly-A tail at the other. Two expansive open reading frames, ORF1a and ORF1b, occupy 2/3 of the whole genome sequence, positioned at the 5′ terminus. The polymeric proteins pp1a and pp1ab, synthesized from the ORFs, undergo proteolytic cleavage by the viral protease into 16 nonstructural proteins (nsps). These nsps are essential in constituting the replication-transcription complexes (RTCs) within double-membrane vesicles (DMVs).^3,4^ In addition, these nsps also execute a variety of functions. For instance, nsp1 participates in the degradation of cellular mRNA and inhibits both IFN-I signal transduction and host protein translation.^5^ Nsp6 restricts autophagosome expansion,^6^ facilitating viral replication, among other activities.

In the cytoplasm of eukaryotic cells, uncapped RNA is rapidly degraded by 5′–3′ exonuclease. Hence, the cap structure is commonly regarded as a significant sign of the stability of RNA molecules. Coronaviruses are the largest known RNA viruses, and their genomic RNA potentially being the largest RNA molecule present in the cytoplasm. The adaptive evolution of coronaviruses has engineered certain strategies to conceal the 5′ terminal triphosphates inherent in their RNAs. The primary method involves the construction of cap structures, serving to emulate the host’s mRNA and facilitating effective viral replication. A substantial body of evidence suggests that coronaviruses engage in capping processes through conventional manner. This means that within the triphosphate RNA, the phosphodiester bonds between β-phosphate and γ-phosphate are hydrolyzed by RNA triphosphatase (nsp13), resulting in RNA with a diphosphate 5′ terminus (5′-PPN). Subsequently, a guanine nucleoside is enzymatically attached at this site by guanylyltransferase (nsp12), generating the key intermediate (GpppN). The process is completed as GpppN undergoes methylation by N7-methyltransferase (nsp14, N7-MTase) and 2′-O-methyltransferase (nsp16, 2′-O-MTase) in the presence of the methyl donor SAM (S-adenosyl methionine), thereby producing cap-0 structures (^7Me^GpppN) and cap-1 structures (^7Me^GpppN_m_).^7,8^

In recent years, our group has conducted research into the molecular mechanisms underpinning the capping and methylation of coronavirus RNA, and the activity characteristics of N7-MTase and 2′-O-MTase.^9–11^ Moreover, Ray et al., Decroly et al. and Furuichi et al. demonstrated the indispensability of the viral cap structure for both viral replication and viral RNA translation.^8,12,13^ Züst et al., Daffis et al. and our group demonstrated that these viral cap structures play a pivotal role in resisting IFN-mediated antiviral responses and especially in escaping the innate immune response,^14–17^ suggesting that ribose methylation of viral RNA may affect viral pathogenesis. It is significant to note that these extensive studies commonly adopt the strategy of introducing relevant mutations into the crucial active center of the 2′-O-MTase, the K-D-K-E motif. This results in the deactivation of nsp16. The resultant protein with impaired function is for studying the physiological characteristics and importance of 2′-O-methylation. Nevertheless, the K-D-K-E motif displays high conservation across diverse viruses and within mammalian 2′-O-MTase. No natural variants of these motif residues have been identified in nature so far. Consequently, a comprehensive understanding of the 2′-O-methyltransferase, including its physiological significance and the exploration of non-conserved sites, aids in understanding, analyzing, and predicting potential threats posed by relevant viral outbreaks.

In this study, we observed that SARS-CoV-2 exhibited a stronger 2′-O-MTase activity than SARS-CoV, characterized by possessing a more distinct cap sequence-specific manner and a higher efficiency. In addition to K-D-K-E motif, Leu-36, Asn-138 and Ile-153 residues were identified as key determinants contributing to the disparity in 2′-O-MTase activity. We introduced these mutations (Leu^36^→Ile^36^, Asn^138^→His^138^, Ile^153^→Leu^153^, from SARS-CoV-2 to SARS-CoV) into the virus using a secure replicon system (transcription and replication-competent SARS-CoV-2 virus-like-particles, trVLP-WT, trVLP-3Mut). Interestingly, the substitution led to a significant decrease in viral replication, accompanied by a strong upregulation of proinflammatory chemokines in the cell cultures. This induction effect is dependent on the cooperative sensing of cytoplasmic RNA sensor MDA5 and LGP2. We performed an unbiased transcriptome screen in Caco-2 cells using RNA-seq, and confirmed that these mutations induced a heightened innate immune response. Furthermore, we generated a model of SARS-CoV-2 trVLPs-infected K18-hACE2 knock-in (KI) mice based on the Lentivirus-N transduction. We thus demonstrated that recombinant SARS-CoV-2 lacking of 2′-O-methylation on a proportion induced a poorer replication efficiency and more severe diseases in vivo. These findings suggested that natural mutations in nsp16 residues other than K-D-K-E motif may confer a potential risk by impacting viral virulence and triggering a robust inflammation response. The varying levels of 2′-O-methylation modifications have a significant impact on immune and inflammatory responses, therefore, ascertaining the optimal regulation of 2′-O-methylation is vital in the design of more efficient, less immunogenic mRNA vaccines.

## Results

### A stronger 2′-O-MTase activity of SARS-CoV-2 than SARS-CoV

**Fig. 1:**
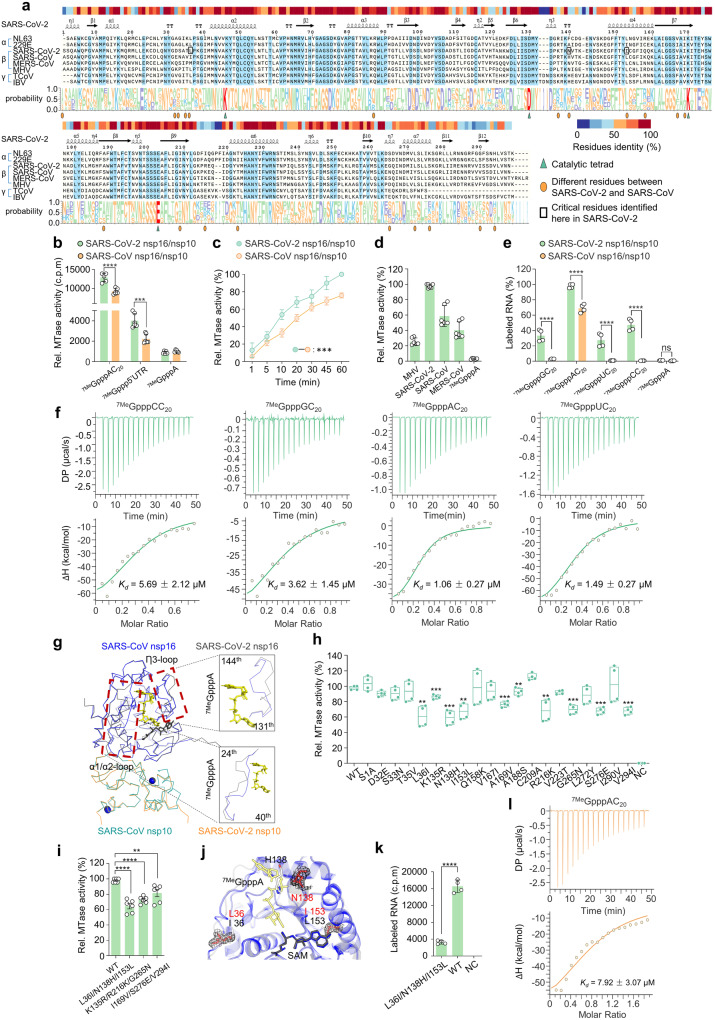
Biochemical and structural mechanisms underpinning the stronger 2′-O-MTase activity of SARS-CoV-2. **a** MUSCLE alignment is performed on representative α-, β-, and γ-coronaviruses nsp16 amino acid sequences and is presented in both text form (middle) and graphical form (bottom), with sequence identity marked at the top of the text. The sequence motif logo is generated using WebLogo. Amino acid characteristics are differentiated by color in the sequence logo. Basic amino acids are represented in blue, acidic amino acids in purple, polar uncharged amino acids in yellow, and non-polar amino acids in green. The K-D-K-E motif is colored by red. **b** The contrast between SARS-CoV-2 (green) and SARS-CoV (orange) regarding 2′-O-MTase activity under the optimal conditions. An excess amount of nsp10 was introduced into the reaction (*n* = 6, mean values ± SD). **c** A comparison was conducted on the capping efficiency between SARS-CoV-2 and SARS-CoV with complete excess of RNA and SAM substrates. The reaction process was terminated at 1, 5, 10, 20, 30, 45 or 60 min by the addition of a 10-fold excess of 20 mM SAH. The values obtained at the established optima were normalized to 100% (*n* = 4, mean values ± SD). **d** Quantitative assessment of 2′-O-MTase activity on cap-0 structures within RNA sequences representing the 5′-terminal untranslated region (5′UTR) of diverse coronaviruses was performed. ^7Me^GpppA was employed as a negative control (*n* = 6, mean values ± SD). **e** The binding affinities of SARS-CoV-2 or SARS-CoV nsp16/nsp10 toward RNAs are measured with different ^3^H-labeled RNA substrates (*n* = 4, mean values ± SD). **f** The ITC profiles demonstrating the dissociation constant (*K*_d_) of ^7Me^GpppCC_20_, ^7Me^GpppGC_20_, ^7Me^GpppAC_20_ and ^7Me^GpppUC_20_ RNA to SARS-CoV-2 nsp16/nsp10 complex. **g** The structures of SARS-CoV-2 nsp16/nsp10 (PDB ID 6WKS, nsp16, gray, nsp10, orange) and SARS-CoV nsp16/nsp10 (PDB ID 3R24, nsp16, blue, nsp10, green) are shown as a ribbon and compared in superposition. Elements displaying variant conformations are systematically highlighted and annotated. SAM (black) and ^7Me^GpppA (yellow) are depicted as a stick model. **h**, **i** The MTase activity of the nsp16 mutants were determined. The activity value for WT was defined as 100%. The data are presented as mean ± standard deviation, derived from four (**h**) or six (**i**) independent experiments. **j** Modeling the location of the 36^th^, 138^th^, and 153^th^ residues in the nsp16 of SARS-CoV-2 (red) or SARS-CoV (colored by atoms, C: white, H: white, N: blue, O: red). These specified residues of SARS-CoV-2 is depicted in the 2Fo-Fc map, contoured at 1.5 σ. **k** Both the WT and the triple mutant were subjected to determining their RNA-binding affinities, using radioactive RNAs as indicators (*n* = 4, mean values ± SD). **l** ITC profiles for the binding of ^7Me^GpppAC_20_ to the triple mutant. The data underwent statistical analysis employing two-way ANOVA with subsequent Tukey’s test (**b**, **e**), unpaired Student’s *t* test (**h**, **i**, **k**), or multiple regression (**c**). The Familywise error rates (FWERs) were computed utilizing the Holm method (**c**). ns not significant, *p < 0.05, **p < 0.01, ***p < 0.001, ****p < 0.0001

Several researches have indicated that the K-D-K-E motif serves as the catalytic active center of 2′-O-MTase among various viruses and determines the enzymatic activity.^10,18,19^ Multiple-sequence comparisons revealed that the K-D-K-E motif exhibited highly conserved across various viruses, including SARS-CoV-2 (Fig. 1a and Supplementary Fig. S1a). Alterations in its sequence arbitrarily led to the inactivation of nsp16 (Supplementary Fig. S1b). However, despite sharing the same catalytic center (Fig. 1a and Supplementary Fig. S1c), a comparison of enzymatic reaction kinetics under optimal conditions revealed that SARS-CoV-2 nsp16/nsp10 exhibited a stronger and more efficient 2′-O-MTase activity than SARS-CoV (Fig. 1b, c and Supplementary Fig. S1d–g). To elucidate the underlying factors contributing to this disparity, we conducted a comprehensive biochemical characterization. In SARS-CoV, the initial nucleotide (^7Me^GpppA) serves as a requisite element for the commencement of methylation by nsp16/nsp10.^10^ Notably, SARS-CoV-2 nsp16/nsp10 can methylate not only ^7Me^GpppA-capped RNA but also ^7Me^GpppG-capped RNA (Supplementary Fig. 1h). The thin-layer chromatography (TLC) showed that SARS-CoV-2 nsp16/nsp10 catalyzed the conversion of the cap-0 structure on ^7Me^GpppA-capped RNA and ^7Me^GpppG-capped RNA to a cap-1 structure (Supplementary Fig. 1i). These results suggested that SARS-CoV-2 nsp16/nsp10 functions in more unique cap-sequence-specific manner than that of SARS-CoV. The similar observation was also validated using the LC-MS-based technical approach.^20^ Moreover, it was found that SARS-CoV-2 nsp16 exhibited robust 2′-O-MTase activity when the 5′-terminal untranslated regions (5′UTRs) of a variety of coronaviruses were used as substrates (Fig. 1d). We carried out an RNA-binding assay and pulled down radiolabeled RNAs to characterize RNA-binding capacity of proteins. Interestingly, it was observed that ^7Me^GpppN-capped RNAs (N, A, G, C, U) could effectively bind the SARS-CoV-2 nsp16/nsp10 complex (Fig. 1e), although not all of them could undergo methylation (Supplementary Fig. 1h); in contrast, SARS-CoV showed no such binding capability. We also analyzed the thermodynamic parameters for the interaction of nsp16/nsp10 complex with ^7Me^GpppN-capped RNA by isothermal titration calorimetry (ITC) (Fig. 1f). The titration curves describing the dissociation constant (*K*_*d*_) showed that ^7Me^GpppA-capped RNA exhibited the highest binding affinity, while the remaining RNA displayed a moderate affinity. In addition to ^7Me^GpppA-capped RNA, the thermodynamic changes did not show exothermic binding upon the addition of ^7Me^GpppN-capped RNA to the SARS-CoV nsp16/nsp10 complex (data not shown).^10^ A previous study indicated that RNA with a 5-nucleotide extension is required as a substrate for SARS-CoV nsp16,^21^ while RNA with a 2-nucleotide extension is adequate for produce 2′-O-MTase activity in SARS-CoV-2 (Supplementary Fig. 1i). Overall, these fundings suggested that SARS-CoV-2 nsp16 exhibited an extensive range of RNA substrate adaptability and binding capacity. We thus expected to reveal its mechanisms of this phenomenon from the crystal structure and sought to verify this using biochemical experiments.

The nsp16/nsp10 complex possesses two asymmetric polypeptide chains, in which chain A (nsp16) and chain B (nsp10) form heterodimers that are stabilized by the formation of protein complexes on the interacting surfaces.^22^ Structural investigation based on the superimposition of SARS-CoV (PDB ID 3R24) and SARS-CoV-2 (PDB ID 6WKS) nsp16/nsp10 interfaces showed an obvious conformational difference in the α1/α2 loop and ƞ3 loop, resulting in a tighter RNA-binding pocket in SARS-CoV-2 (Fig. 1g). A similar conformational difference was observed for structural superpositions of nsp16/nsp10 with the same ligand (PDB ID 6XKM and 3R24, Supplementary Fig. 1k). The optimization of the structure may contribute to the stronger 2′-O-MTase activity of SARS-CoV-2. We assessed that the known mutations spanned the whole protein sequence, with a total of 20 differences by alignment: Ser-1, Asp-32, Ser-33, Thr-35, Leu-36, Lys-135, Asn-138, Ile-153, Gln-158, Val-167, Ile-169, Ala-188, Cys-209, Arg-216, Val-223, Gly-265, Lys-272, Ser-276, Ile-290 and Val-294 (Fig. 1a). Site-directed mutagenesis of these residues from SARS-CoV-2 nsp16 into SARS-CoV nsp16 was carried out and the mutants were studied in vitro. The outcome showed that substitution of the Leu-36, Asn-138, Ile-153, Lys-135, Arg-216, Gly-265, Ile-169, Ser-276 and Val-294 residue respectively resulted in the 2′-O-MTase activity decreasing to varying degrees (Fig. 1h). Further combinatorial analysis and screening of several residues revealed that the L36I/N138H/I153L mutant displayed the most severe reduction in 2′-O-MTase activity (Fig. 1i). This implied that Leu-36, Asn-138 and Ile-153 were the key residues responsible for the difference in terms of 2′-O-MTase activity. The crystal structures showed that Leu-36 and Asn-138 were located on the surface of the RNA-binding pocket, while Ile-153 was located on the binding pocket deeply. These residues played a decisive role in the conformational change (Fig. 1g, j). It was speculated that these residues might be involved in RNA substrate recognition to regulate the methylation process. To confirm our conjecture, we carried out RNA-binding assay and ITC test. As expected, the L36I/N138H/I153L mutant inhibited RNA substrate binding to nsp16/nsp10 (Fig. 1k, l), suggesting that the diversity in 2′-O-MTase activity between SARS-CoV-2 and SARS-CoV primarily arose from different abilities of RNA molecules binding. The presence of these critical residues in SARS-CoV-2 nsp16 significantly enhanced the RNA binding affinity of 2′-O-MTase and broadened its versatility toward a wider range of RNA substrates.

### Determinants of viral replication

Based on the information of SARS-CoV-2 provided by Nextstrain,^23^ Outbreak,^24^ and the Global Initiative on Sharing Avian Influenza Data (GISAID),^25^ we traced the historical evolutionary trajectory of nsp16 mutations from 2020 to 2023. Identity analysis revealed that the similarity of nsp16 sequences from around the world over a span of 3 years was nearly 100% compared with the original sequence (hCoV-19/Wuhan/WIV04/2019, EPI_ISL_402124) (Supplementary Fig. 2a). A chart displaying mutations of nsp16 residues during SARS-CoV-2 evolution showed that although 72 sites experienced mutations, the overall mutation prevalence remained low (Supplementary Fig. 2b). The highest mutation prevalence was observed in A1575V in ORF1b (A178V in nsp16), which was detected in ~4.98% of Beta variant (B.1.351). Moreover, the analysis of incidence frequency (IF) and the phylogenetic tree of SARS-CoV-2 indicated that the residues L36 and N138 remained unchanged during evolution (Supplementary Fig. 2c, d). In February 2022, a substitution of residue 153 from Isoleucine to Valine (I153V) was found in the BA.1 variant; however, IF of the I153V mutation was less than 1% and subsequently disappeared in later variants. These findings suggested that the critical residues of nsp16 (Leu-36, Asn-138, and Ile-153) are highly conserved during virus evolution and have potentially important contributions to viral fitness and replication.

**Fig. 2:**
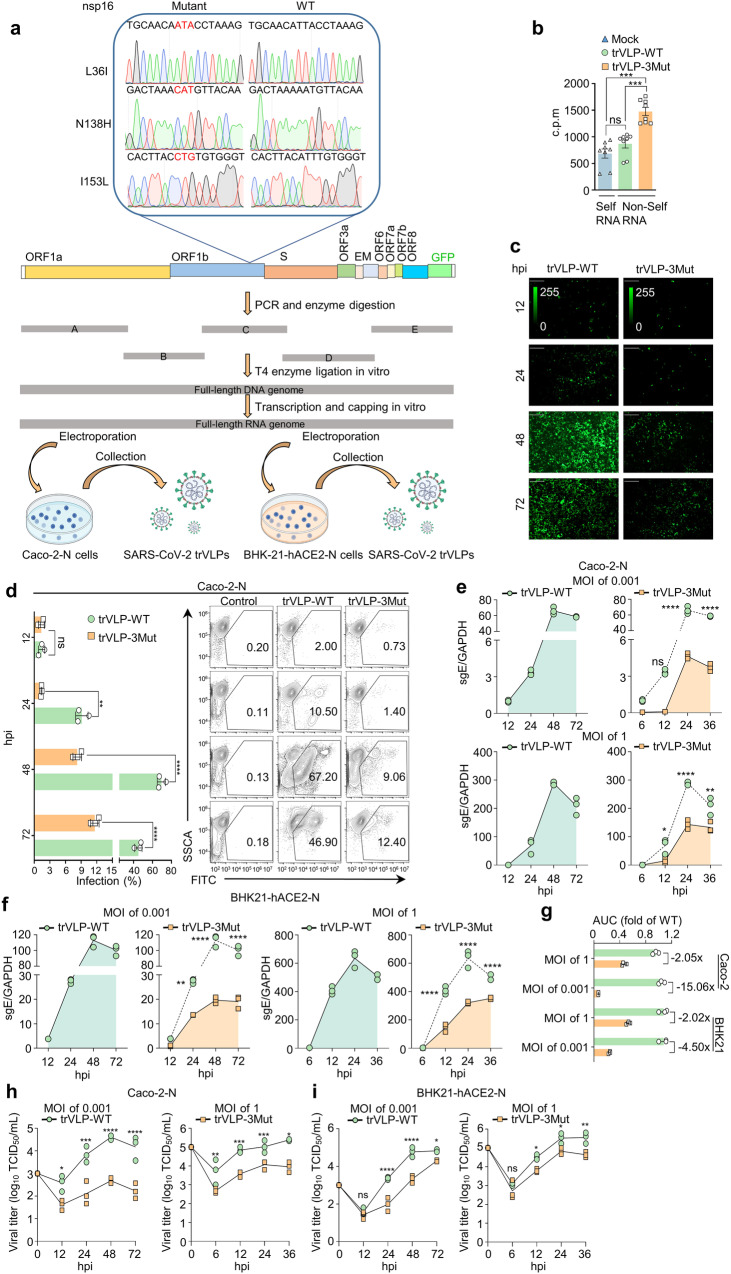
The nsp16 L36I/N138H/I153L mutant inhibited SARS-CoV-2 replication. **a** Flow diagram of replication subsystem construction to obtain SARS-CoV-2 virus-like-particles. The top figure represented the sequences analysis of WT and mutant amino acids of SARS-CoV-2 nsp16 (Leu^36^ → IIe^36^, L36I, N138H and I153L; N, Asn; H, His; I, IIe; L, Leu). Images of cell culture dishes and viruses were produced with BioRender.com. **b** Viral mRNA (Non-Self RNA) was isolated from cells infected with trVLPs at a MOI of 0.01. The MTase activity was assessed using VP39, with cellular RNA (Self RNA) serving as the control. Fluorescence microscopy analysis (**c**) and flow cytometry analysis (**d**) of Caco-2 cells infected with trVLP-WT or trVLP-3Mut at a MOI of 0.001 at 12 hpi, 24 hpi, 48 hpi and 72 hpi. Positive infections with representative (green) images are shown (**c**). Scale bars, 500 μm. Cells were collected and subsequently examined to quantify GFP expression using flow cytometry analysis (**d**). At the designated time points, Caco-2 cells (**e**) or BHK21 cells (**f**) were harvested following infection with trVLPs at a MOI of 0.001 or 1. Subgenomic RNA of the envelope gene (sgE) levels were quantified using qRT-PCR with GAPDH serving as the internal control. **g** The robustness of sgE production from (**e**, **f**) was determined by AUC analysis. **h**, **i** Viral replication kinetics of trVLP-WT or trVLP-3Mut in Caco-2 cells (**h**) or BHK21 cells (**i**) after infection at a MOI of 0.001 or 1 determined using the TCID_50_ assay. Statistical analysis was performed using unpaired Student’s *t* test (**b**, *n* = 8, mean value ± SD) or two-way ANOVA followed by Tukey’s test (**d**–**i**, *n* = 3, mean value ± SD). ns not significant, *p < 0.05, **p < 0.01, ***p < 0.001, ****p < 0.0001

To identify the effect of the deficiency in 2′-O-MTase activity on SARS-CoV-2 infection and replication, the mutants (trVLP-nsp16 L36I, trVLP-nsp16 N138H, trVLP-nsp16 I153L, and trVLP-nsp16 L36I/N138H/I153L, trVLP-3Mut) was constructed on the basis of the infectious clone of trVLP-WT (Fig. 2a), as described previously.^26^ The findings of fluorescence signal, qRT-PCR and Western blot suggested that all trVLPs were successfully constructed in Caco-2 cells that are stably transfected with SARS-CoV-2 N gene (Supplementary Fig. S3a–c). The introduction of mutations was verified via RT-PCR and sequencing (Fig. 2a and Supplementary Fig. S3d, e). We also successfully methylated viral mRNA extracted from infected cells with commercial 2′-O-MTase (vaccinia virus VP39) in vitro (Fig. 2b and Supplementary Fig. 3f). This confirmed the defect of 2′-O-MTase activity was associated with nsp16 mutants in vivo. Caco-2–N cells underwent infection with the trVLP-WT and trVLP-3Mut at a multiplicity of infection (MOI) of 0.001, respectively. The fluorescent signals and infected cell count increased during 12 h post-infection (hpi) to 72 hpi, as determined using fluorescence microscopy and flow cytometry analysis. The trVLP-WT exhibited rapid propagation in Caco-2 cells, infecting 67.20% and 46.90% cells at 48 hpi and 72 hpi, respectively, as opposed to 9.02% and 12.4% for trVLP-3Mut (Fig. 2c, d). Concurrently, viral sgRNA copy numbers in both Caco-2 cells and BHK21 cells were counted using qRT-PCR (Fig. 2e, f) and the viral titers were determined by TCID_50_ (Fig. 2h). The fundings showed that the replication of trVLP-3Mut was severely attenuated in Caco-2 cells and BHK21. The mutant virus exhibited significantly reduced levels of infectious viral particles in comparison to the WT virus. Quantitative analysis using the area under the curve (AUC) for SARS-CoV-2 subgenomic RNA (sgRNA) revealed that compared to trVLP-WT, trVLP-3Mut replicated 2.05-fold (MOI of 1) and 15.06-fold (MOI of 0.001) less efficiently in Caco-2 cells, 2.02-fold (MOI of 1) and 4.50-fold (MOI of 0.001) less efficiently in BHK21 cells (Fig. 2g). Additionally, a single substitution of residue in nsp16 was sufficient to decrease viral 2′-O-MTase activity and inhibit viral replication, further highlighting the crucial role of these residues in nsp16 (Supplementary Fig. S3f–h). Collectively, these findings indicated that the mutation of the Leu-36, Asn-138 and Ile-153 residues, which resulted in a partial deficiency in 2′-O-MTase activity of SARS-CoV-2 nsp16, generated significant decrease in viral replication.

### Significant upregulation of interferon (IFN)-mediated inflammatory responses

**Fig. 3:**
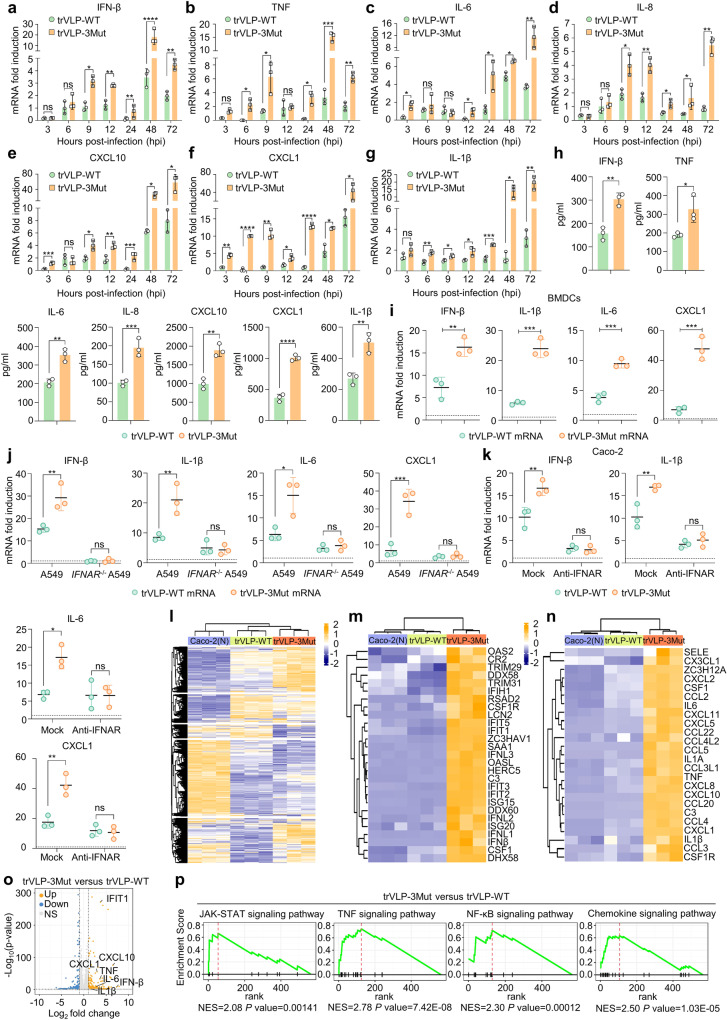
Significant upregulation of IFN pathways and inflammatory pathways was observed in Caco-2 cells infected with trVLP-3Mut. The kinetics for the expression of mRNAs encoding IFN-β (**a**), TNF (**b**) and proinflammatory chemokines (**c**–**g**) in Caco-2 cells which were challenged with trVLP-WT or trVLP-3Mut at a MOI of 0.01 (*n* = 3, mean value ± SD). **h** The concentrations of IFN-β and a spectrum of proinflammatory cytokines within the supernatants of BHK21 cells were quantified by ELISA 24 h post-infection with either trVLP-WT or trVLP-3Mut at a MOI of 0.5 (*n* = 3, mean value ± SD). **i**, **j** Viral mRNA was isolated and purified from Caco-2 cell supernatant 36 h post-infection with trVLP-WT or trVLP-3Mut at a MOI of 0.1. Subsequently, 2 μg of purified viral mRNA was transfected into BMDCs (**i**) or wild-type A549 and *IFNAR*^*−/−*^ A549 cells (**j**). Expression of mRNA encoding IFN-β and corresponding proinflammatory cytokines were assessed 24 h after transfection (*n* = 3, mean values ± SD). **k** Expression of IFN-β and corresponding proinflammatory cytokines 24 h post-infection with trVLPs (MOI of 0.5) in 40 μg/ml neutralizing antibody-pretreated for 2 h or untreated Caco-2 cells (*n* = 3, mean values ± SD). **l** A heatmap illustrates the DEGs in cells infected with trVLP-WT or trVLP-3Mut (fold change$\, > \,$2, *p*$\, < \,$0.05). Heatmaps revealing DEGs in innate immune response signaling pathway (**m**) and inflammatory signaling pathway (**n**). **o** Volcano plot indicating significantly upregulated (orange), downregulated (blue) and nonsignificant genes (gray) between trVLP-WT versus trVLP-3Mut groups. **p** GSEA enrichment plot for genes belonging to the JAK–STAT signaling pathway, TNF signaling pathway, NF-κB and chemokine signaling pathway for trVLP-WT versus trVLP-3Mut groups. Normalized enrichment scores (NESs) were depicted. The dotted lines denoted the RNA copies from the mock (**i**–**k**). The data were statistically analyzed using two-way ANOVA followed by Turkey’s test (**a**–**g**, **j**, **k**) or unpaired Student’s *t* test (**h**, **i**). ns not significant, *p < 0.05, **p < 0.01, ***p < 0.001, ****p < 0.0001

To investigate the causes of replication differences associated with trVLP-WT and trVLP-3Mut, we checked the expression kinetics of mRNAs encoding IFN-β and various proinflammatory cytokines in the context of the first 72 h of infection. The trVLP-3Mut induced a multifold increase in IFN-β, tumor necrosis factor (TNF), proinflammatory chemokines and cytokines compared with trVLP-WT (Fig. 3a–g). We further assessed the expression levels of cytokines associated with early viral infection using enzyme-linked immunosorbent assay (ELISA) (Fig. 3h). These results suggested that trVLP-3Mut initiated significantly higher levels of proinflammatory chemokines and cytokines than trVLP-WT, despite the substantial attenuation of trVLP-3Mut replication (Fig. 2).

To evaluate the role of 2′-O-methylation in the induction of proinflammatory cytokines, RNA containing different 5′-terminal structures (pppRNA, ^7Me^Gppp-capped RNA, and ^7Me^GpppA_m_-capped RNA) were transfected into bone marrow-derived dendritic cells (BMDCs). IFN-β and several proinflammatory cytokines were induced considerably by pppRNA, whereas they were only marginally triggered by ^7Me^GpppA_m_-capped RNA (cap-1) (Supplementary Fig. S4a). The results suggested that 2′-O-methylation played a crucial role in suppressing IFN-I and proinflammatory cytokines expression. We further extracted viral mRNA from the supernatant of Caco-2 cells infected with trVLPs, and transfected them into BMDCs or Caco-2 cells using the same amount. As shown in Fig. 3i and Supplementary Fig. S4b, trVLP-3Mut mRNA effectively resulted in upregulation of IFN-β and proinflammatory cytokines, which indicated a direct correlation between 2′-O-MTase activity and the induction of proinflammatory cytokines. To investigate whether nsp16 or its mutants could directly trigger IFN-I and proinflammatory cytokines, we transfected the nsp16 or mutant nsp16 into BMDCs cells. Both did not yield substantially different amounts of IFN-I or proinflammatory cytokines in BMDCs (Supplementary Fig. S4c, d). Neither nsp16 nor the mutant could inhibit IFN-I and proinflammatory cytokines expression in Sendai virus (SeV)-infected BMDCs. We also verified this phenomenon in HEK 293T cells employing a dual fluorescence reporter system (Supplementary Fig. S4e). To delve into the mechanisms underlying the heightened inflammatory responses triggered by trVLP-3Mut, we investigated whether suppressing the interferon pathway would influence proinflammatory cytokines expression. For this, we transfected the viral mRNA into both WT A549 cells and A549 cells with knockout of the IFN-I receptor (*IFNAR*^*−/−*^), and observed that various proinflammatory cytokines driven by trVLP-3Mut is substantially attenuated in *IFNAR*^*−/−*^A549 cells (Fig. 3j). A similar phenomenon was also observed in IFN-deficient Vero cells (Supplementary Fig. S4f). Furthermore, Ruxolitinib,^27^ an inhibitor that can block IFN signaling pathway, and IFNAR neutralizing antibody which has been verified to inhibit IFN-I receptor signaling in vitro and vivo,^28^ were used. As shown in Fig. 3k and Supplementary Fig. S4g, treatment with Ruxolitinib or IFNAR neutralizing antibody resulted in a reduction of proinflammatory chemokines and cytokines induced by trVLP-3Mut infection, restoring them to the levels observed in trVLP-WT. Interestingly, neither drug treatment nor antibody treatment eliminated the proinflammatory cytokines expression triggered by trVLPs, indicating SARS-CoV-2-mediated multifactorial inflammatory responses. These findings strongly suggested that the excessive production of proinflammatory cytokines triggered by trVLP-3Mut infection was due to uncontrolled IFN-I.

To further evaluate the variations in the innate immune responses in trVLP-3Mut-infected cells, the transcriptome profiles of Caco-2–N cells were analyzed using RNA-seq. Samples were analyzed for correlation and clustered using hierarchical clustering and the Pearson correlation coefficient as a metric (Supplementary Fig. S5a, b). The results showed that the sequencing data were strongly correlated within the same group. We plotted a heatmap for all the differentially expressed genes (DEGs) (Fig. 3l). Differential gene expression analysis of trVLP-WT- and trVLP-3Mut-infected cells identified 694 DEGs; of these, 249 (35.9%) were upregulated and 445 (64.1%) were downregulated (Supplementary Fig. S5c), indicating significant differences in the overall transcriptional performance. We paid special attention to transcripts in the innate immune response and inflammatory response. Based on the heatmap (Fig. 3m, n) and volcano plots (Fig. 3o) summarizing both the expression fold change and the statistical significance, we found that multiple genes related to cellular antiviral innate immunity were significantly upregulated with trVLP-3Mut infection, including IFN-β, TNF, interleukin 6 (IL-6), interleukin 1 beta (IL-1β), C-X-C motif chemokine ligand (CXCL) members and so on. Concurrently, we conducted Gene Ontology (GO) functional enrichment and KEGG pathway analyses to compare the trVLP-3Mut and trVLP-WT groups. The outcomes proved that the pathways enriched by upregulated genes mainly included the “innate immune response”, “inflammatory response”, “TNF signaling pathway”, “viral protein interaction” and so on (Supplementary Fig. S6a, b). On the other hand, gene set enrichment analysis (GSEA) certified that a significant upregulation of JAK-STAT signaling pathway, TNF signaling pathway, nuclear factor kappa B subunit (NF-κB) signaling pathway and chemokine signaling pathway characterized cells infected with trVLP-3Mut (Fig. 3p). The RNA-seq data supported the qRT-PCR results for the associated expression of mRNA encoding IFN and proinflammatory cytokines in trVLP-3Mut-infected cells. In summary, SARS-CoV-2 trVLP-3Mut partially deficient in 2′-O-methylation significantly heightened IFN responses and IFN-mediated inflammatory responses.

### The comparison of transcriptome landscape features

**Fig. 4:**
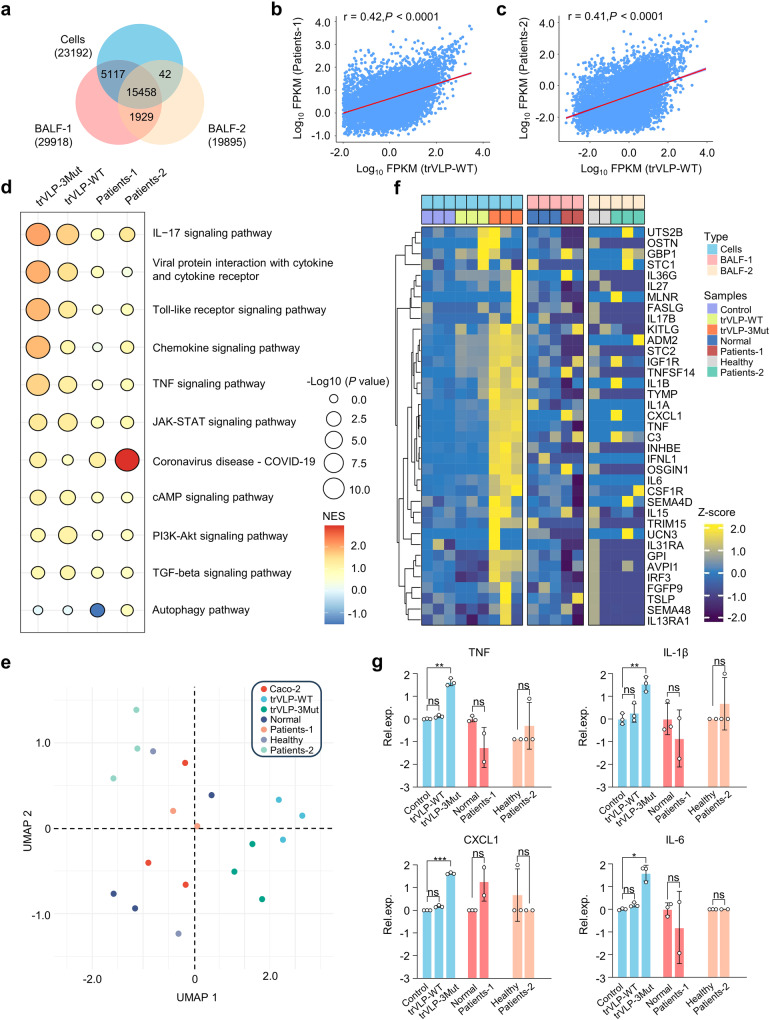
RNA-seq analysis of Caco-2 cells infected with trVLPs revealed conserved and unique transcriptome profiling features compared to bronchoalveolar lavage fluid (BALF) specimens from COVID-19 patients. **a** Venn diagram showing shared mRNA transcripts between Caco-2 cells (Cells) infected with trVLPs and BALF specimens of COVID-19 patients obtained from multiple datasets (BALF-1 and BALF-2). Only orthologous genes expressed in all samples were included in the analysis. **b**, **c** Correlation analysis of FPKM was conducted for trVLP-WT (from Cells dataset, 3) and Patients-1 (from BALF-1 dataset, 2), as well as for trVLP-WT and Patients-2 (from BALF-2 dataset, 3). **d** Bubble plot visualization of GSEA for pathways enriched in trVLP-WT, trVLP-3Mut, Patients-1 and Patients-2. Color coding represents normalized enrichment scores (NES), and bubble sizes correspond to −log_10_ (*p* values). **e** Uniform Manifold Approximation and Projection (UMAP) of the transcriptional data based on genes associated with inflammatory and innate immune responses. **f** Heatmap representation of immune-response-related genes, ordered by hierarchical clustering. **g** The relative expression levels of listed proinflammatory cytokines in both cells and patients groups were shown. The data were statistically analyzed using Pearson’s chi-squared test (**b**, **c**) or two-way ANOVA followed by Turkey’s test (**g**). ns not significant, *p < 0.05, **p < 0.01, ***p < 0.001, ****p < 0.0001

We compared the previously reported multiple transcriptome sequencing data of BALF specimens obtained from COVID-19 patients (project number: PRJCA002273 and CRA002390) with our RNA-seq data.^29,30^ The comparison showed a significant overlap (Fig. 4a), suggesting similar gene expression profiles. Global gene expression analysis revealed a positive correlation (*r* = 0.42) between the transcriptional profiles of trVLP-WT and Patients-1 (from BALF-1 dataset), as well as between the transcriptional profiles of trVLP-WT and Patients-2 (from BALF-2 dataset, *r* = 0.41) (Fig. 4b, c). We normalized the transcriptome data of these samples and constructed a heatmap of the relative expression levels displaying shared genes (Supplementary Fig. S7a). The landscape representation presented a visual summary showing differential expression of overlapping genes. We then performed a GSEA. As shown in Fig. 4d, the “IL-17 signaling pathway” were the most significantly enriched in trVLP-3Mut groups, suggesting a strong association between the inflammatory response driven by trVLP-3Mut and IL-17 family members. The gene signatures in trVLP-3Mut, compared with either Patients-1 or Patients-2, were enriched for pathways associated with the stimulation of ISGs and additional inflammatory response, such as “Toll-like receptor signaling pathway”, “Chemokine signaling pathway”, and “TNF signaling pathway”. Intriguingly, the pathways involved in the cell growth, proliferation, and metabolism in trVLP-3Mut were also significantly upregulated, including the “PI3K-Akt signaling pathway”, “TGF-beta signaling pathway”, and “cAMP signaling pathway”. This unexpected observation requires further investigation.

By conducting Uniform Manifold Approximation and Projection (UMAP) on genes associated with inflammatory and innate immune responses, we observed a wide dispersion between the trVLP-3Mut clusters and Patients clusters (Fig. 4e). This suggested that trVLP-3Mut exhibits unique transcriptomic profiles in immune and inflammatory responses, which were distinct from the BALF samples. Furthermore, multiple gene expression analysis showed that trVLP-3Mut exhibited distinct gene expression patterns in innate and inflammatory pathway (Fig. 4f). Specifically, upregulation of genes involved in IFN and TNF signal transduction, such as interferon regulatory factor 3 (IRF-3), Tripartite motif-containing protein 15 (TRIM15), interferon lambda 1 (IFN-λ1), and tumor necrosis factor superfamily member 14 (TNFSF14) in trVLP-3Mut. Additionally, various transcripts of proinflammatory cytokines, such as TNF, IL-1β, IL-6, and CXCL1, were also upregulated in trVLP-3Mut, but not in the trVLP-WT and Patients specimens (Fig. 4g). On the other hand, we established protein-protein interaction (PPI) networks from DEGs in trVLP-3Mut, and utilized a confidence score of 0.9 to generate dominant networks including several subnetworks for a comprehensive understanding of the paradigm of interaction of immune and inflammatory pathways induced by trVLP-3Mut (Supplementary Fig. S7b). In summary, these results demonstrated the correlation between trVLP-WT infection-directed cellular events and SARS-CoV-2-mediated multiple biological processes, thereby highlighting previously unrecognized differences both in innate immune and inflammatory responses driven by trVLP-3Mut.

### SARS-CoV-2 trVLPs infection in K18-hACE2 KI mice based on Lentivirus-N transduction

We sought to further validate these findings in vivo. To enable the mice to express SARS-CoV-2 N, 6-to 8-week-old K18-hACE2 KI mice were intranasally inoculated with the Lentivirus encoding for SARS-CoV-2 N (Lenti-N) (Supplementary Fig. S8a). After intranasal inoculation with 2 × 10^7^ or 2 × 10^9^ copies of Lenti-N virion, mice did not lose weight (Supplementary Fig. S8b). We examined the N expression in lung tissue of the mice and observed that N expression peaked at 14 days post-infection (dpi) (Supplementary Fig. S8c). Viral RNA was detected in the trachea, lung, and turbinate, which may be related to the delivery and expression mode of lentivirus (Supplementary Fig. S8d). Especially, we also detected high levels of hACE2 RNA in multiple tissues and organs, indicating that infection of Lenti-N did not affect the expression of hACE2 (Supplementary Fig. S8e). Further immunofluorescence staining of lung section suggested that robust viral N protein and hACE2 were expressed successfully in K18-hACE2 KI mice (Supplementary Fig. S8f).

**Fig. 5:**
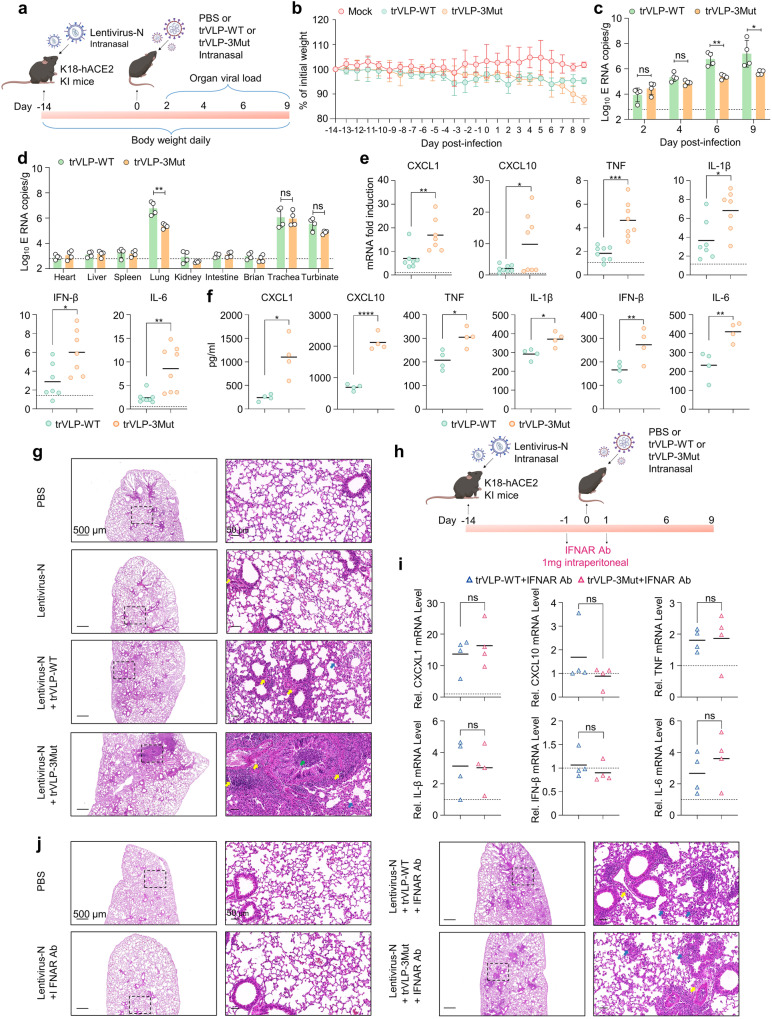
SARS-CoV-2 trVLPs infection in Lentivirus-N transduced K18-hACE2 KI mice. **a** A scheme of administration of SARS-CoV-2 N and trVLPs in K18-hACE2 KI mice. Six-to eight-week-old K18-hACE2 KI mice were inoculated with Lentivirus-N (10^9^ copies, intranasal route), trVLP-WT (4 × 10^6^ TCID_50_, intranasal route), trVLP-3Mut (4 × 10^6^ TCID_50_, intranasal route) or PBS mock as indicated. **b** Mouse weight change was monitored after trVLPs infection (*n* = 5 per group). **c** qRT-PCR was employed to measure the levels of viral RNA in the lung tissues of mice following Lentivirus-N transduction and trVLPs inoculation at indicated days (*n* = 4 per group). **d** The distribution of SARS-CoV-2 viral RNA across different tissues was assessed at 6 dpi following the administration of trVLPs (*n* = 4 per group). **e** Expression of mRNAs encoding related cytokines and chemokines in lung tissues of mice, at 6 days post-infection (dpi) with trVLP-WT or trVLP-3Mut, were determined (*n* = 4 per group). **f** IFN-β and various proinflammatory cytokines of BALF were measured at 6 dpi with trVLP-WT or trVLP-3Mut using ELISA (*n* = 4 per group). **g** H&E staining was carried out on lung tissues obtained from K18-hACE2 mice after intranasal administration of PBS (Mock), Lentivirus-N, Lentivirus-N + trVLP-WT and Lentivirus-N + trVLP-3Mut at 6 dpi (scale bars, 500 μm or 50 μm) illustrating inflammatory cell infiltration (yellow arrow), alveolar septal thickening (blue arrow), and bronchiolar inflammatory exudates filling (green arrow). Each histological image served as a representative sample for a cohort of three mice. **h** A scheme of IFNAR neutralizing antibody treatment. K18-hACE2 KI mice were treated with an IFNAR neutralizing antibody through intraperitoneal injection both 24 h before and 24 h after trVLPs infection. **i** Expression of relevant cytokines and chemokines at 6 dpi with trVLPs in antibody-treated mice (*n* = 4 per group). **j** Representative images of H&E staining of pathological tissue damage in the lungs of PBS (Mock), Lentivirus-N + IFNAR antibody, Lentivirus-N + trVLP-WT + IFNAR antibody, and Lentivirus-N + trVLP-3Mut + IFNAR antibody at 6 dpi (scale bars, 500 μm or 50 μm). Images of the mice and viruses were created with BioRender.com (**a**, **h**). The dotted lines denoted the RNA copies from mock-infection mice (**c**–**e**, **i**). The data were expressed as mean ± standard deviation, and statistical analyses were performed using two-way ANOVA Turkey’s multiple comparison (**c**, **d**) or unpaired Student’s *t* test with Welch’s correction (**e**, **f**, **i**). ns not significant, *p < 0.05, **p < 0.01, ***p < 0.001, ****p < 0.0001

To maximize trVLPs replication, K18-hACE2 KI mice were inoculated with 4 × 10^6^ TCID_50_ of trVLPs via an intranasal route at 14 days after Lenti-N transduction (Fig. 5a). The trVLP-WT-infected mice had slight weight loss compared with control mice. In contrast, the mice infected with trVLP-3Mut exhibited more weight loss during the 7–9 dpi (Fig. 5b). SARS-CoV-2 S protein and sgE RNA were identified using Western blot and qRT-PCR (Supplementary Fig. S9b, c), indicating that trVLPs were successfully rescued in lungs of K18-hACE2 KI mice. As expected, in the case of a consistent effect of N transduction (Supplementary Fig. S9d), we observed a strong decrease in trVLP-3Mut replication in lung tissues after intranasal inoculation at 6 dpi or 9 dpi compared with WT (Fig. 5c). Notably, detectable viral RNA was found not only in lungs, but also in trachea, turbinate tissues (Fig. 5d). This phenomenon possibly related to the natural tropism of the SARS-CoV-2.^31^ Consistent with what was observed in vitro, a strong induction of mRNAs encoding multiple cytokines and chemokines was determined in lung homogenates at 6 dpi from trVLP-3Mut-infected K18-hACE2 KI mice (Fig. 5e). Concurrently, an elevated expression of proinflammatory chemokines and cytokines was observed in the BALF of mice infected with trVLP-3Mut, as determined by ESLISA (Fig. 5f). Hematoxylin and eosin (H&E) staining indicated that mice transduced with Lenti-N alone presented mild immune cell infiltration at perivascular, peribronchiolar, and alveolar locations compared with mock at 6 dpi. Lenti-N-transduced, trVLP-WT-infected mice showed an increase in visible lung injury, featuring alveolar septal thickening, neutrophil and lymphocyte accumulation in perivascular and bronchiole peribronchiolar locations. In contrast, trVLP-3Mut-infected mice developed interstitial pneumonia characterized by large amount of infiltrating inflammatory cells, thickening of the alveolar septal or collapse of the alveolar structure, and distinctive inflammatory exudates filling in bronchioles (Fig. 5g). The finding indicated trVLP-3Mut has acquired the capability to induce a substantial increase in lung injury compared with trVLP-WT. To test whether the lung injury and increased pathogenicity driven by trVLP-3Mut were caused by uncontrolled IFN responses, antibodies for IFNAR were administered during trVLPs infection. As shown in Fig. 5i and Supplementary Fig. S9e, the antibody treatment effectively eliminated the differential expression of proinflammatory chemokines and cytokines caused by trVLP-WT and trVLP-3Mut infections. In addition, examination of lung tissue through H&E staining revealed comparable disease progression in mice infected with trVLP-WT and trVLP-3Mut. The specific manifestations observed included a clear structure of the bronchus, absence of any apparent inflammatory exudate in the lumen, increased infiltration of immune cells around the blood vessels, and thickening of the alveolar septal accompanied by accumulation of alveolar epithelial cells (Fig. 5j). These results confirm that the heightened pathogenicity induced by trVLP-3Mut was a result of uncontrolled IFN-I response. Overall, these results strongly indicated that SARS-CoV-2 trVLP-3Mut exhibited a poorer replication efficiency and caused more severe diseases in vivo than trVLP-WT.

### The immunological recognition of SARS-CoV-2 RNA affected by 2′-O-methylation

**Fig. 6:**
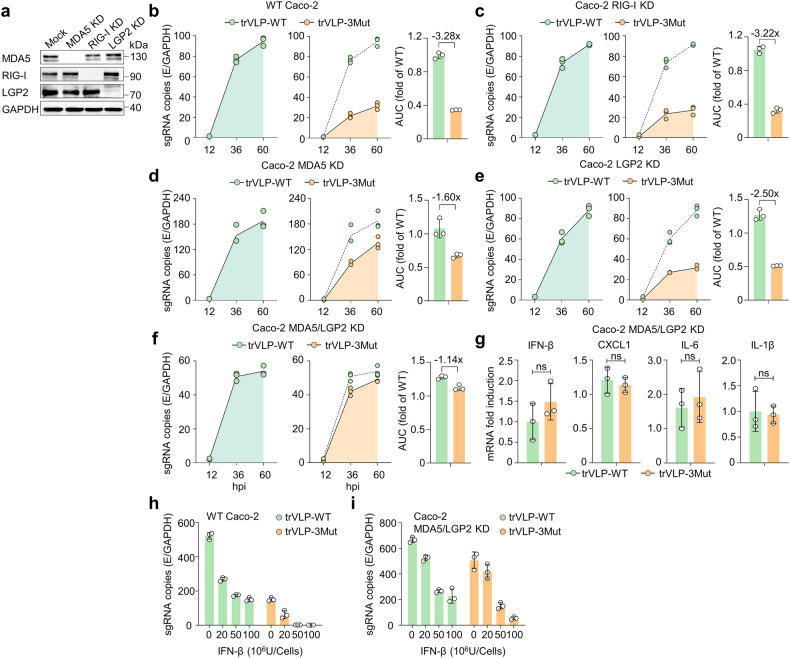
MDA5/LGP2-dependent upregulation of inflammatory responses in trVLP-3Mut-infected Caco-2 cells. **a** SiRNA-mediated knockdown (KD) efficiency was confirmed using Western blot. **b**–**f** The siRNA-transfected Caco-2 cells were infected with trVLP-WT or trVLP-3Mut at MOI of 0.01. Cell lysates were harvested to quantify sgE RNA by qRT-PCR. Robustness of sgE production from (**b**) to (**f**) was depicted as AUC. The data presented represented the mean derived from three separate experiments, each performed in duplicate. **g** Expression of IFN-β and related proinflammatory cytokines were quantified by qRT-PCR in knockdown of MDA5 and LGP2 genes of Caco-2 cells infected with trVLP-3Mut or trVLP-WT at 36 hpi (MOI of 0.01). The data were the average of three experiments performed in duplicate. SARS-CoV-2 sgRNA copy numbers were quantified at 36 hpi in WT Caco-2 cells (**h**) or siRNA of MDA5/LGAP2-transfected Caco-2 cells (**i**), which were pretreated with the indicated doses of IFN-β for 4 h before being infected with trVLP-WT or trVLP-3Mut (MOI of 0.5). The data are representative of the mean ± SD from three experiments, each conducted in duplicate. Statistical analysis was carried out using two-way ANOVA followed by Tukey’s test; ns not significant, *p < 0.05, **p < 0.01, ***p < 0.001, ****p < 0.0001

Previous researches have showed that SARS-CoV-2 genome can be detected by the retinoic acid-inducible gene I (RIG-I)-like receptors (RLRs). The RLRs family comprises three members: RIG-I, melanoma differentiation- associated protein 5 (MDA5) and laboratory of genetics and physiology 2 (LGP2). To investigate the correlation between RLRs expression and the restriction of trVLP-3Mut infection, we transiently silenced target genes by delivering small interfering RNAs (siRNAs) into the Caco-2 cells, respectively. Detectable silencing of target genes was assessed by Western blot (Fig. 6a). The viral sgE copies in cells challenged with trVLP-3Mut was significantly reduced by 3.28-fold and 3.22-fold comparing with those challenged with trVLP-WT, as determined by AUC analysis (Fig. 6b, c). Knockdown of RIG-I proteins in Caco-2 cells did not significantly alter the replication difference between trVLP-WT and trVLP-3Mut. By contrast, trVLP-3Mut replicated 1.60-fold and 2.50-fold less efficiently compared with trVLP-WT after MDA5 knockdown or LGP2 knockdown, respectively (Fig. 6d, e). Silencing MDA5 and LGP2 simultaneously, the sgRNA level of trVLP-3Mut was diminished by only 1.14-fold compared with that of trVLP-WT (Fig. 6f). Furthermore, compared to trVLP-WT, trVLP-3Mut induced an increased expression of IFN-β and proinflammatory chemokines and cytokines (Fig. 3a–g), which was reduced in MDA5/LGP2-silenced Caco-2 cells (Fig. 6g). We observed a higher restriction of trVLP-3Mut replication, indicating an increased sensitivity to IFN-β treatment in Caco-2 cells infected with trVLP-3Mut rather than trVLP-WT (Fig. 6h). Of interest, in contrast to trVLP-WT, trVLP-3Mut was barely detectable after 4 h of pretreatment of Caco-2 cells with 50–100 units (U) of IFN-β. However, pretreatment involving a minimum of 100 U of IFN-β was required to limit trVLP-3Mut replication in MDA5/LGP2-silenced Caco-2 cells (Fig. 6i). This revealed that the induction of type I interferon, facilitated by MDA5 and LGP2, directly influenced the restriction of trVLP-3Mut replication. Further investigation is required to understand how MDA5 and LGP2 specifically generate downstream signal transduction and cascade amplification effects associated with interferon pathway, rather than inflammation pathway. Together, these results suggested that the restriction of 2′-O-methylation-deficient viral replication was dependent on the cooperative sensing of MDA5 and LGP2.

## Discussion

Even though viral cap structures exhibited similarities across different viral taxa, the biochemical mechanisms that form the cap structures are diverse.^7^ For example, Vesicular stomatitis virus (VSV) utilizes a distinctive RNA: GDP polyribonucleotide transferase that facilitates the transfer of a monophosphorylated RNA cap onto GDP.^32^ Influenza A virus (IAV) and influenza B virus (IBV) utilize a strategy known as “cap snatching”, a process whereby the viruses cleave the cap structure of host cellular mRNAs and subsequently append these segments to their viral mRNAs.^33^ In contrast, the capping mechanism of coronaviruses is highly conserved, and the formation of the cap structure is dependent on the continuous action of viral-encoded enzymes, including RNA triphosphatase (nsp13), RNA guanylyl transferase (GTase) (nsp12), N7-methyltransferase (nsp14), 2′-O-methyltransferase (nsp16) and cofactor (nsp10).^7,34^ Among these proteins, 2′-O-MTase has a well-conserved catalytic center (Fig. 1a). Surprisingly, after further assessment, we revealed that SARS-CoV-2 nsp16 exhibited innovative properties, specifically manifesting its function with a unique cap-sequence-specific manner. According to spatial structure of nsp16/nsp10, it can be observed that amino acids changes lead to increased flexibility in the crystal pattern of RNA-binding pockets, enabling conformational changes necessary for enhanced substrate binding and accommodation. Thus, SARS-CoV-2 nsp16 had a stronger 2′-O-MTase activity than SARS-CoV, accompanied by an extensive range of RNA substrate adaptability and binding capacity. Viswanathan et al. also found that the expansion of the RNA-binding pocket allows the N7-methyl guanidine group of cap-0 (^7Me^Go) to bind to the deep groove formed, even enabling the methylation of very short RNA substrates.^35^ These substantial disparities indicated that the 2′-O-methylation of SARS-CoV-2 held paramount biological significance.

Multiple sets of clinical evidence show that COVID-19 and SARS exhibit distinct pathological processes, although the SARS-CoV-2 genome shares 77.5% nucleotide sequence identity with that of SARS-CoV.^36–39^ In terms of mortality, a comprehensive descriptive analysis by the World Health Organization (WHO) indicates that the fatality rate of COVID-19 as of October 2023 was estimated to be 0.9%, which is much lower than that of SARS (9.5%).^40,41^ From an infection-to-onset perspective, an analysis of exposure intervals based on confirmed cases showed an average incubation period of 6.4 days for COVID-19 (interquartile range, 5.6–7.7 days) and 5 days for SARS (interquartile range, 2–10 days).^42,43^ Furthermore, the incubation period for asymptomatic carriers of COVID-19 is estimated to span 1 to 14 days, with a more typical range of 3–10 days. This duration exceeds the incubation period associated with SARS.^40^ From the perspective of the disease course, SARS is more acute, while COVID-19 causes mild or asymptomatic illness in most cases (30–60%). Overall, COVID-19 presents different epidemiological dynamics from SARS, with lower mortality and fever rates, longer median-/asymptomatic incubation period, and milder symptoms. It is clearly imperative to define the virological characteristics underpinning these differences. Combined with the important role of viral RNA 2′-O-methylation in immune escape and the observed experimental results, we hypothesize that the differences between SARS-CoV-2 and SARS-CoV 2′-O-MTase may contribute to their different clinical manifestations. Given the phenotypic discrepancies observed in the 2′-O-MTase mutants, it is tempting to speculate that a stronger 2′-O-MTase activity confers an improved ability to circumvent host innate immune detection. This mechanism results in the host manifesting reduced expression of IFN, ISGs, and proinflammatory cytokines, thereby limiting pathogenesis and affecting the disease process. This change may be a mechanism and strategy adjustment of SARS-CoV-2. We put forward a possible explanation for the early epidemiological differences between COVID-19 and SARS. Differential methylation of viral RNA may be a typical example of escaping host cell pattern recognition and promoting the spread of foreign viral RNA. Our observations corroborate and expand upon the conclusions, which demonstrated that SARS-CoV-2 replication prompts a postponed interferon response.^44^ Our fundings highlighted the unique antagonistic impacts of both SARS-CoV and SARS-CoV-2 on IFN-I production and signaling, influencing disease progression differently. Furthermore, corroborative reports from other research have delineated a subdued cytokine response following SARS-CoV-2 infection. They postulated that this could stem from a viral-mediated obstruction of the STAT1/2 signaling pathway.^45^ Several findings suggest that SARS-CoV-2 has established effective mechanisms to inhibit host IFN’s reduction.^46,47^ These findings, along with our own research results, offer a multitude of perspectives explaining the considerable number of asymptomatic or mildly symptomatic instances encountered throughout the COVID-19 pandemic. In conclusion, SARS-CoV-2 may have developed various evasion strategies to suppress innate immune surveillance, resulting in a different epidemiological profile from SARS-CoV infection. The role of 2′-O-methylation in modulating transmission efficiency and overall disease progression of COVID-19 and SARS needs to be further expanded and studied. In addition, the current mRNA vaccines designed for SARS-CoV-2 primarily utilize cap-1 structures due to immunogenicity constraints.^48,49^ Our findings indicated that varying levels of cap-1 modification have a notable impact on immune and inflammatory reactions. Therefore, determining optimal control over cap-1 modification is crucial for designing more effective and less immunogenic mRNA vaccines, offering valuable insights for future research.

Recent studies showing nsp16 regulating IFN-I-mediated innate immune pathways, by Russ et al. and Schindewolf et al., are similar to our work.^50,51^ They found that defect of SARS-CoV-2 nsp16 function (K-D-K-E mutations) attenuates viral replication in a IFN-I-dependent manner. Mechanistically, they demonstrated the necessity of IFIT-1 and IFIT-3 in mediating nsp16 mutant attenuation. While extensive studies on the correlation between nsp16 and innate immune pathways, particularly those based on K-D-K-E mutations, have rapidly advanced our understanding of the importance of 2′-O-methylation, our current knowledge regarding the roles of other amino acids remains limited. A notable strength of our study is the identification of Leu-36, Asn-138, and Ile-153 as pivotal residues which govern the disparity in 2′-O-MTase activity between SARS-CoV-2 and SARS-CoV, thereby extending our focus beyond the exclusively emphasized K-D-K-E motif. This conclusion is drawn from a systematic comparison in nsp16. Multiple-sequence alignment showed that K-D-K-E motif is highly conserved in a variety of viruses.^10^ Natural mutants in this motif have not been observed in nature. Therefore, exploring other residues would provide a more comprehensive insight into the function and mechanism of 2′-O-MTase in different viruses and even mammals. In addition, although both works mentioned above demonstrated that SARS-CoV-2 harboring a mutation in the K-D-K-E motif is more sensitive to IFN-I than wild-type SARS-CoV-2, our work further clarified that unique residues of SARS-CoV-2 nsp16 affects IFN-mediated inflammatory response. We observed that even partial absence of 2′-O-methylation on Viral RNA was adequate for recognition by the cytoplasmic sensors MDA5 and LGP2, leading to the upregulation of IFN-I and triggering a more robust inflammatory response. Moreover, Russ et al. and Schindewolf et al. show that silencing IFIT-1 or IFIT-3 partially restores fitness to the nsp16 mutant with a lack of 2′-O-methylation in hamsters and in mice; this insight was not encompassed in our present research. This suggests that the restriction of SARS-CoV-2 replication through MDA5-/LGP2-dependent and IFIT-1 mediated mechanisms may represent distinct antiviral pathways. Generally, these results validate the significance of SARS-CoV-2 RNA 2′-O-methylation as a crucial evasion strategy against host immune recognition and resistance to IFN-mediated antiviral response. Natural mutations in critical residues of nsp16 could potentially contribute to future spillover events, highlighting the inherent risk of severe diseases.

One potential limitation of our study was the inability to generate chimeric viruses harboring gain-of-function mutations, as SARS-CoV has demonstrated higher virulence in terms of replication and cellular damage in vitro.^52,53^ Consequently, we adopted a more secure model to comprehensively investigate the functions of viral proteins. It is noteworthy that Lentivirus-N-mediated K18-hACE2 KI mice model successfully recapitulated the infection and replication of SARS-CoV-2 in ABSL-II laboratory, ensuring safety, efficacy, and experimental simplicity. Indeed, our study underscored the pivotal role played by 2′-O-methylation in governing disease severity and immunopathology.

In summary, we comprehensively compared SARS-CoV-2 and SARS-CoV nsp16/nsp10 2′-O-MTase, and identified the crucial residues accountable for their variations. Our findings indicated that mutations other than K-D-K-E motif exerted significant impacts on viral virulence as well as the inflammatory pathways. We provided functional insights regarding the impact of SARS-CoV-2 nsp16 on virulence and pathogenicity.

## Materials and methods

### Radioactive reagents

S-Adenosyl (methyl-^3^H) methionine (67.3 Ci/mmol) and (α-^32^P) GTP (3000 Ci/mmol) were sourced from PerkinElmer.

### Cells

Caco-2 cells, BHK21 cells, HEK-293T cells, Vero cells, A549 cells, A549 *IFNAR*^*−/−*^ cells were cultured with DMEM (Gibco) enriched with 10% (vol/vol) fetal bovine serum (FBS) and 50 U/ml penicillin/streptomycin under the condition of 5% CO_2_ at 37 °C. Through lentivirus infection and resistance screening, Caco-2 cell line and BHK21-hACE2 cell line with stable expression of the N gene was constructed. Bone marrow cells were isolated from the tibia and femur of C57BL/6J mice and then cultured in RPMI-1640 supplemented with 50 ng/ml GM-CSF (Peprotech) for 8 days for bone marrow-derived dendritic cells (BMDCs). All cells are free of mycoplasma.

### Mice

All the B6/JGpt-H11^*em1Cin(K18-ACE2)*^/Gpt mice (K18-hACE2 KI mice, heterozygote, #T037657) were obtained from GemPharmatech Co., Ltd. (Nanjing, China) and were maintained in individually ventilated cages (IVCs) in ABSL-II (AUP # SKLV-AE2023-007). The 6- to 8-week-old mice were infected via intranasal route with indicated Lentivirus, and then were weighed and monitored daily. In subsequent experiments, 14 days after Lentivirus-N transduction, mice were intranasally inoculated with 4 × 10^6^ TCID_50_ of SARS-CoV-2 trVLPs, and the weights of mice were monitored daily. Mice were sacrificed at 2, 4, 6, 9 dpi for tissue processing. For experiments of IFNAR neutralizing antibody treatment, mice received two intraperitoneal injections of 1 mg of MAR1–5A3 IFNAR neutralizing antibody (BioXCell), both 24 h before and 24 h after trVLPs infection. All experiments were made to minimize animal suffering and have been approved by an ethical committee.

### Lentivirus

The construction and preparation of Lentivirus-N was reported previously.^26^ Lentivirus-N and Lentivirus-Vector were concentrated using ultracentrifugation. The copy numbers of lentivirus were quantified via qRT-PCR based on the standard curve.

### Expression and purification of protein

The sequences of nsp16 (YP_009725311) and nsp10 (YP_009742617) acquired from NCBI database were inserted into pET30a vector, respectively. In brief, the corresponding plasmid was transformed into *E. coli* BL21 (DE3). Picked colony was grown in Luria broth medium with kanamycin at 37 °C. When the OD600 of the culture reached 0.6, the culture was induced at 16 °C for 16 h by adding 0.4 mM isopropyl-β-D-thiogalactoside (IPTG). Ultrasonic centrifugation was then performed using an ultrasonic cell disruptor. The cleared lysates were incubated with nickel-nitrilotriacetic acid (Ni-NTA) resin for 1 h. Proteins were eluted with 40 mM Tris–HCl containing 250 mM imidazole. The nsp10 and nsp16 were purified on an Enrich SEC 650 Column (BIO-RAD). The purified proteins were electroporated using SDS-PAGE and then stained by Coomassie blue.

### Preparation of multiple RNA substrates

RNA substrates corresponding to the 5′-UTR sequences of coronaviral genome were transcribed in vitro. Primers containing the T7 promoter (TAATACGACTCACTATA) were designed to initiate the transcription. Two μg of dsDNA template was used for RNA in vitro transcription using HiScribe^TM^ T7 kit (New England BioLabs) according to the manufacturer’s instructions. At the end of reaction, 2 units of DNase I were added to digest the dsDNA template. The in vitro transcribed pppRNA was then extracted with phenol–chloroform. pppAC_n_ is produced by the identical approach.

The GpppA,^7Me^GpppA and ^7Me^GpppA_m_ RNAs were synthesized utilizing the Vaccinia Capping System (New England BioLabs) and commercial VP39 (New England BioLabs). Ten mCi of S-adenosyl (methyl^-3^H) methionine, instead of cold SAM, was employed as the methyl donor for the synthesis of ^3^H-labeled cap substrates (*^7Me^GpppA-RNA or *^7Me^GpppG-RNA). The ^32^P-labeled RNA substrates (G*pppA, ^7Me^G*pppA, ^7Me^G*pppA_m_, ^7Me^G*pppA-RNA, ^7Me^G*pppG-RNA, ^7Me^G*pppC-RNA, and ^7Me^G*pppU-RNA; the asterisks indicate that the following phosphates were α-GTP-^32^P-labeled) were produced as described previously.^9^ The cap analogs, ^7Me^GpppA, were obtained from New England BioLabs.

### Biochemical assays for MTase activity

For ^3^H-methyl-incorporation MTase activity assays of SARS-CoV-2 nsp16/nsp10, 1 μg of proteins and 3 μg of RNA substrates were incubated in a 20 μl reaction containing 50 mM Tris–HCl (pH 7.0), 2 mM DTT, 2 mM ZnCl_2_, 40 units of RNase inhibitor, 0.01 mM SAM, 0.3 μCi of S-adenosyl (methyl-^3^H) methionine at 30 °C for 1 h. In contrast, for MTase activity measures of SARS-CoV nsp16/nsp10, 1 μg of purified proteins and 3 μg of RNA substrates were mixed in a 20 μl reaction containing 40 mM Tris–HCl (pH 7.5), 2 mM DTT, 2 mM MgCl_2_, 40 units of RNase inhibitor, 0.01 mM SAM, 0.3 μCi of S-adenosyl (methyl-^3^H) methionine at 37 °C for 1 h. The ^3^H-labeled RNAs were isolated utilizing DEAE-Sephadex A-50 chromatography columns. Subsequent quantification was performed through liquid scintillation counting (PerkinElmer), consistent with the methodology outlined in prior research.^54^ The c.p.m values represents the number of nuclear decays recorded by the liquid scintillator in 1 min, which can be used to reflect 2′-O-methyltransferase activity.

### TLC

In an 8.5 μl reaction (40 mM Tris–HCl, 2 mM MgCl_2_, 2 mM DTT, 10 units RNase inhibitor, 0.2 mM SAM), 1 μg of proteins and 2000 c.p.m of ^32^P-labeled RNA substrates were incubated at 30 °C for 2 h. Afterward, 0.5 μl nuclease P1 (New England BioLabs) and 1 μl RNase-free water were mixed to release the cap structures. The samples were spotted onto polyethyleneimine cellulose-F plates (Merck), and developed in a solvent system containing 0.4 M ammonium sulfate. The degree of incorporation of the ^32^P-labeled cap into the RNA was ascertained by scanning the TLC chromatogram using Phosphor Imager.

### RNA-binding assay

Three μg of ^3^H-labeled RNA substrates and 4 μg of His_6_-tagged proteins were combined in 100 μl 40 mM Tris–HCl (pH 7.0) containing 2 mM MgCl_2_. The mixture was then shaken by rotation at 4 °C overnight. Subsequently, 20 μl Ni-NTA resin was added into the mixture and gently mixed at 4 °C for 30 min. After discarding the supernatant, the mixture underwent two washes with 40 mM Tris–HCl to eliminate unbound ^3^H-labeled RNA substrates. It was then resuspended in 100 μl of the same buffer. Of this, 30 μl was allocated for Western blot analysis, while the remaining volume was subjected to measurement of radioactive activity via liquid scintillation.^10^

### Isothermal titration calorimetry

We subjected the purified proteins to denaturation/refolding treatment, as previously reported.^55^ ITC was performed with 20 μM nsp16/nsp10 complex and 100 μM RNA using a Malvern MICROCAL PEAQ-ITC machine. Experimental parameters were set as follows: temperature (°C), 25; reference power (μcal/s), 10.0; feedback, high; stir speed (rpm), 750; initial delay (s), 60; injection spacing (s), 150; injection duration (s), 4. Prior to data analysis, the corresponding concentration of RNA solution was injected into 50 mM Tris–HCl alone, which was used as the control. Afterward, the heat of the dilution of RNA was subtracted from the experimental curve to obtain an accurate fitting curve. Data analysis was conducted using MicroCal PEAQ-ITC Analysis Software. The dissociation constant, *K*_*d*_, was accordingly derived.

### Establishment of multiple-sequence alignment and acquisition of protein models

The multiple-sequence alignment was performed and merged with the PDB file using ESPript 3.x,^56^ and visualization was done using WebLogo.^57^ In accordance with the PDB file, PyMOL software was used for further exploration of the conformational characteristics.

### Cloning and assembly of the SARS-CoV-2 genome

In brief, the full-length genome of SARS-CoV-2 (MN908947 strain) was divided into five fragments (A, B, C, D, E), and the fragments were cloned into the pCCI, pMV or pLVX vectors.^26^ The T7 promoter (TAATACGACTCACTATAG) was incorporated at the 5′ terminus of fragment A, and the poly(A) tail was appended to the 3′ terminus of fragment E. The SARS-CoV-2 N gene substituted with GFP. The five fragments were amplified through PCR assays using DNA Polymerase (Takara). Following 1% agarose gel verification, the fragments were purified using the gel extraction kit (Omega). The resultant target product was subjected to digestion by type IIS restriction endonuclease (BsaI for fragments A and B, BsmBI for fragments C, D, and E). Equal molar number of digested fragments were ligated overnight at 4 °C using T4 ligase (New England BioLabs) to obtain full-length cDNA.

### RNA transcription and electroporation

The full-length template and N gene were transcribed in vitro using the mMESSAGE mMACHINE T7 Transcription Kit (Thermo Fisher Scientific) as described previously.^58^ A total of 20 μg of full-length mRNA and 10 μg of N mRNA were added into a 4 mm cuvette (Bio-Rad) containing 0.4 ml of Caco-2–N cells (8 × 10^6^) in Ingenio Electroporation Solution (Mirus). A single electrical pulse was applied using a GenePulser apparatus (Bio-Rad) set at 270 V with a pulse length of 30 ms at 950 μF. The mixture was placed at room temperature for 5 min and then cultured in a T-75 flask for 3 days. The supernatant was collected as the virus P0 generation.

### TCID_50_

The trVLP infectivity was quantified in terms of a tissue-culture infectious dose of 50% (TCID_50_) using the endpoint method.^26,59^ The TCID_50_ was calculated following the Reed and Muench method. The AUC was derived from the viral titers measured at various time points, as displayed on the *y*-axis.

### Flow cytometry analysis

Cells were dissociated using trypsin and resuspended in PBS. About 10^4^ cells of each sample were analyzed using a flow cytometry analyzer (BECKMAN COULTER) and FlowJo software.

### Western blot

The cell was washed twice with PBS and lysed using RIPA (Beyotime) containing 1% cocktail. Consequently, whole-cell lysates were obtained via ice lysis for 30 min. A total of 10% SDS loading buffer was added. Afterward, the lysates were electrophorized in polyacrylamide gel and transferred to the nitrocellulose membrane. The membrane was blocked with 5% BSA for 1 h and subsequently incubated with primary antibody for 3 h. The primary antibodies used were indicated as follows: anti-MDA5 (Proteintech, 21775-1-AP), anti-RIG-I (ABclonal, A18003), anti-LGP2 (ABclonal, A8257), anti-GAPDH (Proteintech, 60004-1-lg), anti-SARS-CoV-2 S1 (Sino Biological, 40591-T62), SARS-CoV-2 N (Sino Biological, 40143-MM05), anti-Flag (Cell Signaling Technology, #14793), and anti-hACE2 (Sino Biological, 10108-RP01). Following three washes with TBST, the membranes were incubated with the appropriate secondary antibody. The membranes were finally visualized using the ChemiDoc MP (Bio-Rad).

### qRT-PCR

The cell supernatant was extracted using Trizol LS reagent (Thermo Fisher Scientific). The obtained 1 μg RNA was used to synthesized the cDNA using the HiScript IV RT SuperMix (Vazyme). The mRNA levels of hACE2, N, E, sgE and GAPDH were measured with the specific probes using TaqMan Probe Master Mix (YEASEN). Relative expression levels of the target genes were calculated using the $\Delta \Delta$CT method. The relevant primers and probes were shown in Table S1.

### RT-PCR and sequencing

RNA was extracted using the Trizol reagent. cDNA amplification was performed using One-Step RT-PCR Kit (Invitrogen). The PCR products were subjected to Sanger sequencing, and the results were presented using the SnapGene software.

### RNA-seq

The methods were described previously.^60^ The provided accession number: HRA005976.

### Analysis of NGS RNA-seq data

RNA-seq reads were first trimmed using the FASTX-Toolkit software. The clean reads were then mapped to the human genome (GRCh38) or SARS-CoV-2 genome (NC_045512.2) using HISAT2 with default parameters. Gene expression was calculated using feature counts in the SubReads package (v1.5.3). The expected fragments per kilobase of transcript per million fragments (FPKM) values were computed according to the formula: FPKM = total exon fragments/(uniquely mapped reads × exon length). DEGs were calculated via the DESeq2 package (v 1.26.0) with the following criteria: adjusted *p* value$\, < \,$0.05 and fold-change$\, > \,$2. Functional enrichment analysis was conducted on the list of DEGs with the cluster Profiler package (v 3.14.3). GO and KEGG pathway annotations were downloaded from the Gene Ontology Resource and KEGG databases, respectively. The interacting proteins were identified using STRING (http://string-db.org/) with an interaction score parameter height confidence of 0.9. For an enhanced graphical representation, the PPI subnetwork visualization was accomplished with Cytoscape version (3.5.0).

### Comparison between cells and patient RNA-seq datasets

The analysis procedure is consistent with that described earlier. The mean value of the controls corresponding to the cell and BALF samples was first calculated. Then, we used the scale function of the base package (R 4.1.0) to standardize the cell and BALF data with the “center” parameter or the mean value calculated for the control group. The merge function was used to integrate the cell and BALF data. The enrichment plots and heatmaps were generated in R using the ClusterProfiler package or ComplexHeatmap, respectively.

### Histopathological analysis

The mice were euthanized, and their lung tissues were subsequently collected. The lung tissues were then fixed with 4% formalin, progressively dehydrated, and finally embedded in paraffin. Each embedded tissue was sectioned into 3 μm thickness longitudinal sections. The tissue sections were stained with H&E. The extent of pulmonary damage was assessed by meticulously evaluating several parameters: the degeneration of alveolar epithelial cells, the distension of parenchymal walls, the manifestation of edema and hemorrhage, and the infiltration of inflammatory cells.

### Immunofluorescence assay

The sections were incubated with anti-SARS-CoV-2 N antibody (Sino Biological, 1:200) and anti-hACE2 antibody (Cell signaling Technology, 1:200). Following washing with PBS buffer (pH 7.4) on a decolorization shaker, the sections were incubated with corresponding secondary antibodies (Servicebio, 1:200) in darkness for 50 min at room temperature. Afterward, the sections were counterstained with DAPI (Beyotime). After drying, the sections were sealed with Antifade Mounting Medium (Beyotime), and scanned using a Pannoramic MIDI system (3DHISTECH).

### Capture of viral mRNA

Viral mRNA was captured and isolated from the cell supernatants 48 hpi at a MOI of 0.1 using the mRNA purification kit (New England BioLabs). The purified mRNA can be utilized for subsequent experiments involving transfection and MTase activity assay.

### ELISA

Following trVLPs infection, the cell culture supernatants or BALF of mice were collected and the concentrations of TNF, CXCL10, CXCL1, IL-6, IL-8, IL-1β and IFN-β were quantified employing ELISA in adherence to the protocol (BioLegend).

### Supplementary information

Supplementary Materials for Natural evidence of coronaviral 2'-O-Methyltransferase activity affecting viral pathogenesis via improved substrate RNA binding

## Data Availability

Transcriptome data of BALF of COVID-19 patients were sourced from the BIG Data Center (https://bigd.big.ac.cn/), retrievable via the accession numbers CRA002390 and HRA000143. Our RNA-seq data have been recorded in the GSA database, with the accession number HRA005976. Sequences of nsp16 were downloaded from the NCBI database (https://www.ncbi.nlm.nih.gov/), including NL63, 229E, SARS-CoV-2, SARS-CoV, MERS-CoV, MHV, TCoV, IBV, Vaccinia virus, and Flavivirus, as well as the mammalian 2′-O-methyltransferase Fstj. The whole genome sequence information of SARS-CoV-2 (MN908947.3 strain) used to replicon construction came from the NCBI database. Global information of diverse SARS-CoV-2 variants have been retrieved from Nextstrain (https://nextstrain.org/), Outbreak (https://outbreak.info/), and the GISAID database (https://gisaid.org/). The structural coordinates utilized in this research were accessible via the RCSB Protein Data Bank (https://www.rcsb.org) under the accession codes 3R24, 6WKS, and 6XKM. Unprocessed data can be accessed from the corresponding author without restrictions.

## References

[1] Zhu N (2020). A novel coronavirus from patients with pneumonia in China, 2019. N. Engl. J. Med..

[2] Das SK (2020). The pathophysiology, diagnosis and treatment of corona virus disease 2019 (COVID-19). Indian J. Clin. Biochem..

[3] Mousavizadeh L, Ghasemi S (2021). Genotype and phenotype of COVID-19: their roles in pathogenesis. J. Microbiol. Immunol. Infect..

[4] Wang DH (2021). The SARS-CoV-2 subgenome landscape and its novel regulatory features. Mol. Cell.

[5] Tanaka T, Kamitani W, DeDiego ML, Enjuanes L, Matsuura Y (2012). Severe acute respiratory syndrome coronavirus nsp1 facilitates efficient propagation in cells through a specific translational shutoff of host mRNA. J. Virol..

[6] Cottam EM, Whelband MC, Wileman T (2014). Coronavirus NSP6 restricts autophagosome expansion. Autophagy.

[7] Chen Y, Guo D (2016). Molecular mechanisms of coronavirus RNA capping and methylation. Virol. Sin..

[8] Decroly E, Ferron F, Lescar J, Canard B (2011). Conventional and unconventional mechanisms for capping viral mRNA. Nat. Rev. Microbiol..

[9] Chen Y (2009). Functional screen reveals SARS coronavirus nonstructural protein nsp14 as a novel cap N7 methyltransferase. Proc. Natl Acad. Sci. USA.

[10] Chen Y (2011). Biochemical and structural insights into the mechanisms of SARS coronavirus RNA ribose 2’-O-methylation by nsp16/nsp10 protein complex. PLoS Pathog..

[11] Chen Y (2013). Structure-function analysis of severe acute respiratory syndrome coronavirus RNA cap guanine-N7-methyltransferase. J. Virol..

[12] Furuichi Y, Shatkin AJ (2000). Viral and cellular mRNA capping: past and prospects. Adv. Virus Res..

[13] Ray D (2006). West Nile virus 5’-cap structure is formed by sequential guanine N-7 and ribose 2’-O methylations by nonstructural protein 5. J. Virol..

[14] Zust R (2011). Ribose 2’-O-methylation provides a molecular signature for the distinction of self and non-self mRNA dependent on the RNA sensor Mda5. Nat. Immunol..

[15] Zhang Z (2021). Live attenuated coronavirus vaccines deficient in N7-Methyltransferase activity induce both humoral and cellular immune responses in mice. Emerg. Microbes Infect..

[16] Pan R (2022). N7-Methylation of the coronavirus RNA cap is required for maximal virulence by preventing innate immune recognition. mBio.

[17] Daffis S (2010). 2’-O methylation of the viral mRNA cap evades host restriction by IFIT family members. Nature.

[18] Wilamowski M (2021). 2'-O methylation of RNA cap in SARS-CoV-2 captured by serial crystallography. Proc. Natl Acad. Sci. USA.

[19] Nencka R (2022). Coronaviral RNA-methyltransferases: function, structure and inhibition. Nucleic Acids Res..

[20] Benoni R (2021). Substrate specificity of SARS-CoV-2 Nsp10-Nsp16 methyltransferase. Viruses.

[21] Bouvet M (2010). In vitro reconstitution of SARS-coronavirus mRNA cap methylation. PLoS Pathog..

[22] Rosas-Lemus M (2020). High-resolution structures of the SARS-CoV-2 2’-O-methyltransferase reveal strategies for structure-based inhibitor design. Sci. Signal..

[23] Hadfield J (2018). Nextstrain: real-time tracking of pathogen evolution. Bioinformatics.

[24] Gangavarapu K (2023). Outbreak.info genomic reports: scalable and dynamic surveillance of SARS-CoV-2 variants and mutations. Nat. Methods.

[25] Shu Y, McCauley J (2017). GISAID: global initiative on sharing all influenza data—from vision to reality. Euro Surveill..

[26] Ju X (2021). A novel cell culture system modeling the SARS-CoV-2 life cycle. PLoS Pathog..

[27] Quintas-Cardama A (2010). Preclinical characterization of the selective JAK1/2 inhibitor INCB018424: therapeutic implications for the treatment of myeloproliferative neoplasms. Blood.

[28] King KR (2017). IRF3 and type I interferons fuel a fatal response to myocardial infarction. Nat. Med..

[29] Xiong Y (2020). Transcriptomic characteristics of bronchoalveolar lavage fluid and peripheral blood mononuclear cells in COVID-19 patients. Emerg. Microbes Infect..

[30] Zhou Z (2020). Heightened innate immune responses in the respiratory tract of COVID-19 patients. Cell Host Microbe.

[31] Hassan AO (2020). A SARS-CoV-2 infection model in mice demonstrates protection by neutralizing antibodies. Cell.

[32] Ogino T, Banerjee AK (2007). Unconventional mechanism of mRNA capping by the RNA-dependent RNA polymerase of vesicular stomatitis virus. Mol. Cell.

[33] Tsukamoto Y (2023). Inhibition of cellular RNA methyltransferase abrogates influenza virus capping and replication. Science.

[34] Yan L (2021). Cryo-EM structure of an extended SARS-CoV-2 replication and transcription complex reveals an intermediate state in cap synthesis. Cell.

[35] Viswanathan T (2020). Structural basis of RNA cap modification by SARS-CoV-2. Nat. Commun..

[36] Giovanetti M (2021). Evolution patterns of SARS-CoV-2: snapshot on its genome variants. Biochem. Biophys. Res. Commun..

[37] Kirtipal N, Bharadwaj S, Kang SG (2020). From SARS to SARS-CoV-2, insights on structure, pathogenicity and immunity aspects of pandemic human coronaviruses. Infect. Genet. Evol..

[38] Zhang YZ, Holmes EC (2020). A genomic perspective on the origin and emergence of SARS-CoV-2. Cell.

[39] Zhou P (2020). A pneumonia outbreak associated with a new coronavirus of probable bat origin. Nature.

[40] Chen J (2020). Pathogenicity and transmissibility of 2019-nCoV-A quick overview and comparison with other emerging viruses. Microbes Infect..

[41] Petrosillo N, Viceconte G, Ergonul O, Ippolito G, Petersen E (2020). COVID-19, SARS and MERS: are they closely related?. Clin. Microbiol. Infect..

[42] Varia M (2003). Investigation of a nosocomial outbreak of severe acute respiratory syndrome (SARS) in Toronto, Canada. CMAJ.

[43] Backer JA, Klinkenberg D, Wallinga J (2020). Incubation period of 2019 novel coronavirus (2019-nCoV) infections among travellers from Wuhan, China, 20-28 January 2020. Euro Surveill..

[44] Xia H (2020). Evasion of type I interferon by SARS-CoV-2. Cell Rep..

[45] Miorin L (2020). SARS-CoV-2 Orf6 hijacks Nup98 to block STAT nuclear import and antagonize interferon signaling. Proc. Natl Acad. Sci. USA.

[46] Blanco-Melo D (2020). Imbalanced host response to SARS-CoV-2 drives development of COVID-19. Cell.

[47] Wolfel R (2020). Virological assessment of hospitalized patients with COVID-2019. Nature.

[48] DiPiazza AT (2021). COVID-19 vaccine mRNA-1273 elicits a protective immune profile in mice that is not associated with vaccine-enhanced disease upon SARS-CoV-2 challenge. Immunity.

[49] Han Y (2022). mRNA vaccines expressing homo-prototype/Omicron and hetero-chimeric RBD-dimers against SARS-CoV-2. Cell Res..

[50] Russ A (2022). Nsp16 shields SARS-CoV-2 from efficient MDA5 sensing and IFIT1-mediated restriction. EMBO Rep..

[51] Schindewolf C (2023). SARS-CoV-2 uses nonstructural protein 16 to evade restriction by IFIT1 and IFIT3. J. Virol..

[52] Chu H (2021). SARS-CoV-2 induces a more robust innate immune response and replicates less efficiently than SARS-CoV in the human intestines: an ex vivo study with implications on pathogenesis of COVID-19. Cell. Mol. Gastroenterol. Hepatol..

[53] Chu H (2020). Comparative tropism, replication kinetics, and cell damage profiling of SARS-CoV-2 and SARS-CoV with implications for clinical manifestations, transmissibility, and laboratory studies of COVID-19: an observational study. Lancet Microbe.

[54] Ahola T, Laakkonen P, Vihinen H, Kaariainen L (1997). Critical residues of Semliki Forest virus RNA capping enzyme involved in methyltransferase and guanylyltransferase-like activities. J. Virol..

[55] Lin S (2020). Crystal structure of SARS-CoV-2 nsp10/nsp16 2’-O-methylase and its implication on antiviral drug design. Signal Transduct. Target Ther..

[56] Robert X, Gouet P (2014). Deciphering key features in protein structures with the new ENDscript server. Nucleic Acids Res..

[57] Crooks GE, Hon G, Chandonia JM, Brenner SE (2004). WebLogo: a sequence logo generator. Genome Res..

[58] Li, Y. et al. An optimized high-throughput SARS-CoV-2 dual reporter trans-complementation system for antiviral screening in vitro and in vivo. *Virol. Sin*. 10.1016/j.virs.2024.03.009 (2024).10.1016/j.virs.2024.03.00938548102

[59] Lindenbach BD (2009). Measuring HCV infectivity produced in cell culture and in vivo. Methods Mol. Biol..

[60] Wang H (2023). NSUN2-mediated M(5)c methylation of IRF3 mRNA negatively regulates type I interferon responses during various viral infections. Emerg. Microbes Infect..

