# Supplementary material for: Natural evidence of coronaviral 2′-O-methyltransferase activity affecting viral pathogenesis via improved substrate RNA binding
Source: Signal Transduct Target Ther. 2024 May 29;9:140. doi: 10.1038/s41392-024-01860-x (PMC11137015; doi:10.1038/s41392-024-01860-x)
Supplement: Supplementary file 1 — Supplementary Materials for Natural evidence of coronaviral 2'-O-Methyltransferase activity affecting viral pathogenesis via improved substrate RNA binding [file 41392_2024_1860_MOESM1_ESM.docx]

Supplementary Materials for

Natural evidence of coronaviral 2'-O-Methyltransferase activity affecting viral pathogenesis via improved substrate RNA binding

Jikai Deng^1,5^, Shimin Yang^1,5^, Yingjian Li^1^, Xue Tan^1^, Jiejie Liu^1^, Yanying Yu^2^, Qiang Ding^2^, Chengpeng Fan^3^, Hongyun Wang^1^, Xianyin Chen^1^, Qianyun Liu^1^, Xiao Guo^1^, Feiyu Gong^1^, Li Zhou^1,4^, Yu Chen^1^*

^1^State Key Laboratory of Virology, RNA Institute, College of Life Sciences and Frontier Science Center for Immunology and Metabolism, Wuhan University, Wuhan, China.

^2^School of Medicine, Tsinghua University, Beijing, China.

^3^School of Basic Medical Sciences, Wuhan University, Wuhan, China.

^4^Animal Bio-Safety Level III Laboratory/Institute for Vaccine Research, Wuhan University School of Medicine, Wuhan, China.

^5^These authors contributed equally to this work.

*Corresponding author: Yu Chen, State Key Laboratory of Virology, College of Life Sciences, Wuhan University, Wuhan, 430072, P. R. China. E-mail: chenyu@whu.edu.cn

**This PDF file includes:**

Fig. S1 to S9

Table S1

**
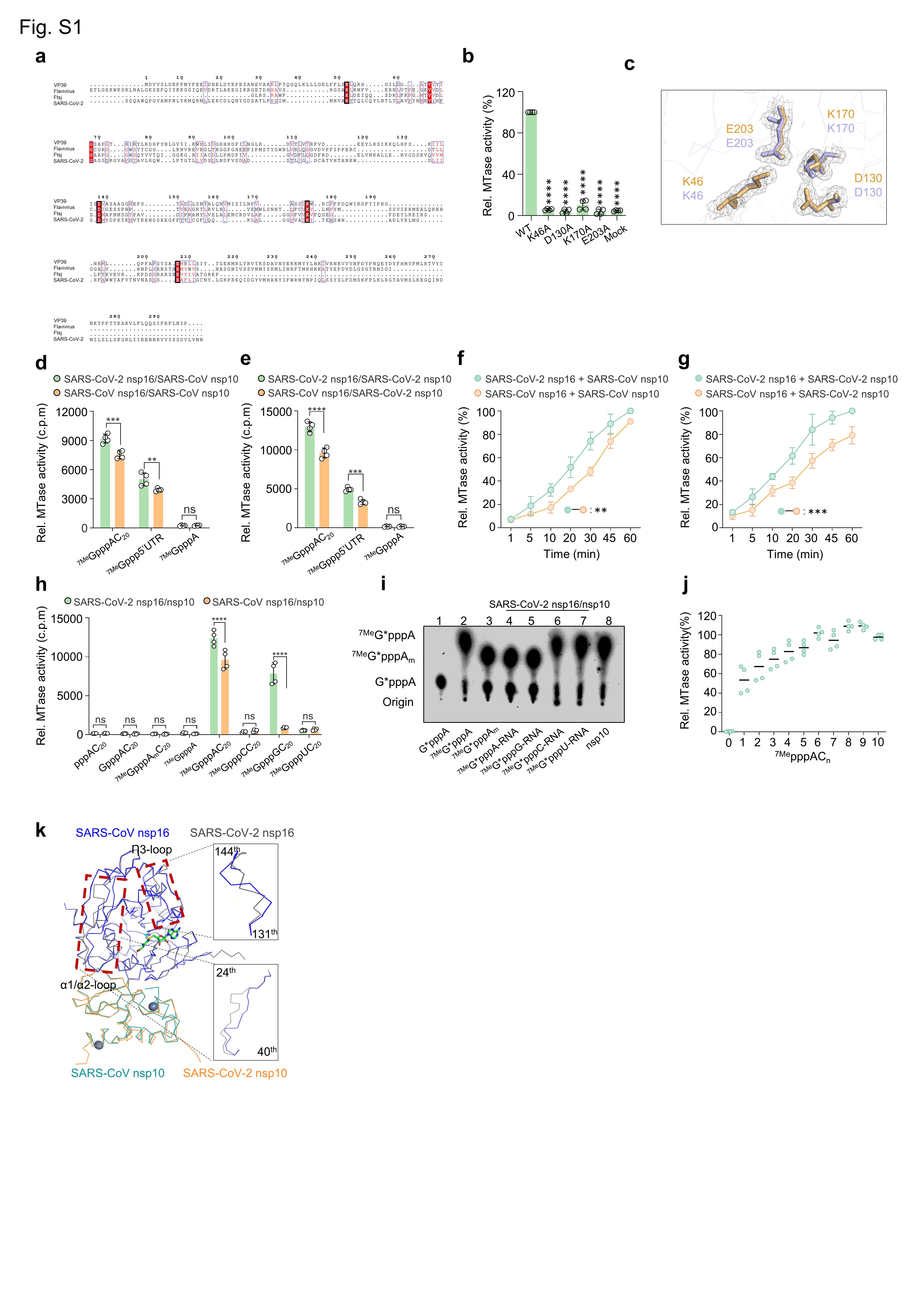
Fig. S1. RNA-binding affinity analysis of SARS-CoV-2 nsp16/nsp10 complex**. (**a**) Multiple-sequence alignment of nsp16 amino acid sequences across various virus. The K-D-K-E catalytic tetrad is highlighted in black boxes. (**b**) Related MTase activity of SARS-CoV-2 nsp16 mutants in K-D-K-E motif were detected using ^7Me^GpppAC_20_ as the substrate in ^3^H-methyl-incorporation assays. An activity of 100% corresponds to that of the WT (n = 4, mean values ± SD). (**c**) The locations of the 46^th^, 130^th^, 170^th^, 203^th^ residues in SARS-CoV-2 nsp16 (orange) and SARS-CoV nsp16 (blue) are labeled as sticks. The 2Fo-Fc electron density map is shown for K-D-K-E motif in black mesh, contoured at 1.5 σ. (**d, e**) Different nsp10 (**d**, SARS-CoV nsp10; **e**, SARS-CoV-2 nsp10) were used to detect the MTase activity of SARS-CoV-2 nsp16 and SARS-CoV nsp16 under optimal conditions. Equal amounts of nsp16 were incubated with 1 μg ^7Me^GpppAC_20_, ^7Me^Gppp 5'UTR or ^7Me^GpppA in the presence of ^3^H-labeled SAM and an absolute excess of nsp10. The values at determined optima were set to 100% (n = 4, mean values ± SD). (**f, g**) Different nsp10 (**f**, SARS-CoV nsp10; **g**, SARS-CoV-2 nsp10) were used to assess the capping efficiency of SARS-CoV-2 nsp16 or SARS-CoV nsp16 in the presence of a complete excess of RNA and SAM substrates. The reaction was stopped at 1, 5, 10, 20, 30, 45 or 60 min by diluting the reaction mixture with a 10-fold excess of 20 mM ice-cold SAH. The values at determined optima were set to 100% (n = 4, mean values ± SD). (**h**) Different RNA substrates (1 μg of each) were incubated with SARS-CoV-2 nsp16/nsp10 or SARS-CoV nsp16/nsp10 for 1h under optimal conditions, and radioactivity incorporation was detected via liquid scintillation counting (n = 4, mean values ± SD). (**i**) The TLC assay using different ^32^P-labeled RNA substrates (lanes 4–7). The positions of origin and migration of G*pppA, ^7Me^G*pppA and ^7Me^G*pppA_m_ (lanes 1, 2 and 3) are indicated on the left as molecular markers. The nsp10 group was used as a negative control (lane 8). (**j**) The effect of RNA substrates of different lengths (^7Me^GpppAC_n_, n = 0-10) on 2'-O-MTase activity (n = 4, mean values ± SD). Values at optimum RNA lengths were arbitrarily set to 100%. (**k**) The structures of SARS-CoV-2 nsp16/nsp10 (PDB ID 6XKM, gray) with SAM and SARS-CoV nsp16/nsp10 (PDB ID 3R24, blue) with SAM are shown as ribbons and compared in superposition. Elements with variant conformations were highlighted and marked. SAM are depicted as a stick model and colored by atoms (C: green, O: red, N: blue, H: white, S: orange). The data were statistically analyzed using unpaired Student’s t test with Welch’s correction (**b**) or two-way ANOVA followed by Turkey’s test (**d, e, h**). Statistically significant differences versus SARS-CoV-2 nsp16 + SARS-CoV nsp10 and SARS-CoV nsp16 + SARS-CoV nsp10 or SARS-CoV-2 nsp16 + SARS-CoV-2 nsp10 and SARS-CoV nsp16 + SARS-CoV-2 nsp10 through time points were determined by multiple regression (**f, g**). Familywise error rates (FWERs) were calculated using the Holm method. ns, not significant, **P* < 0.05, ***P* < 0.01, ****P* < 0.001, *****P* < 0.0001.

**
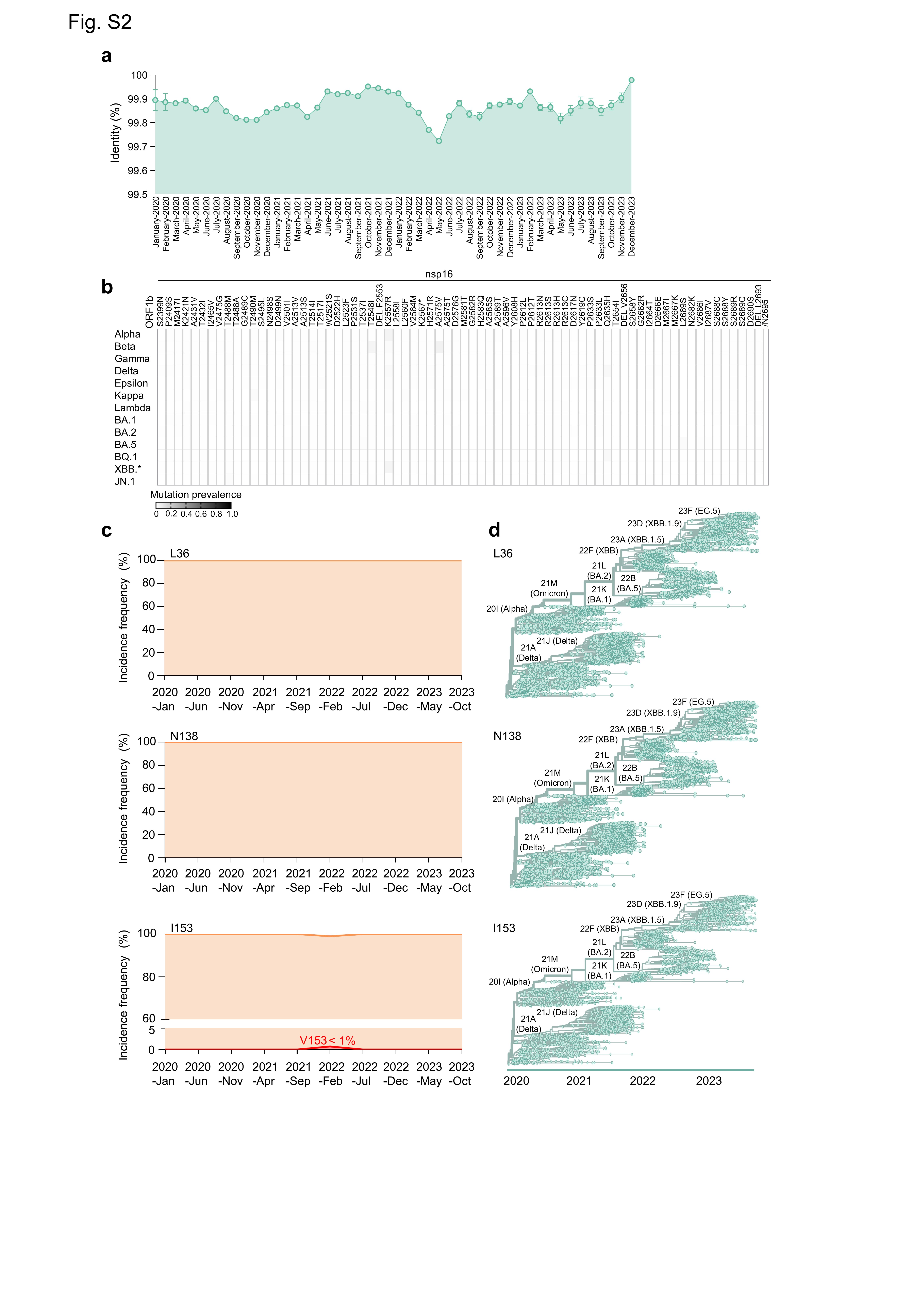
Fig. S2.** **Evolutionary trajectory of the nsp16 mutations.** (**a**) Sequence identity statistics of nsp16 mutations compared to the original nsp16 in each month worldwide from 2020 to 2023. The data source was acquired and curated from the GISAID database. (**b**) Mutation prevalence across the most prevalent lineages globally over the 3 years. The data source was obtained from Outbreak. (**c**) Incidence frequency (IF) of the 36^th^, 138^th^, and 153^th^ residues of SARS-CoV-2 nsp16 from 2020 to 2023. The data source was acquired from Nextstrain. (**d**) Phylogenetic tree of SARS-CoV-2 strains associated with variants of concern (VOCs), obtained from Nextstrain. The visualization is colored by the residues L36, N138, and I153 in nsp16.

**
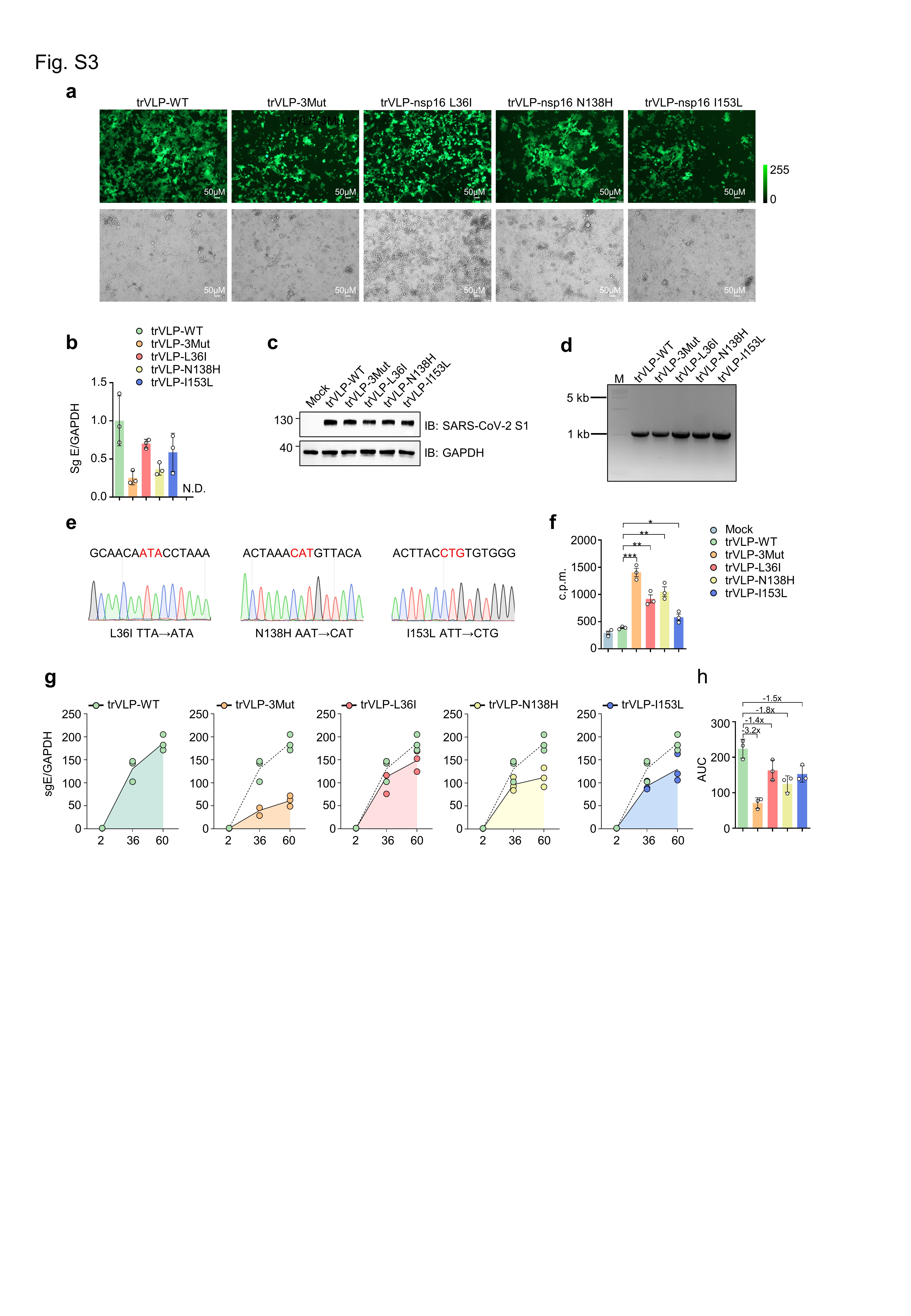
Fig. S3. Generation of SARS-CoV-2 trVLPs.** (**a**) Fluorescence microscopy analysis of P0-trVLP-infected cells. Representative trVLP-positive (green) and light images are shown. (**b**) The total RNAs of P0-virus were extracted and qRT-PCR assays were conducted to determine viral sgRNA levels. Error bars represented the standard deviations from one of three independent experiments performed in triplicate. N.D., no detection. (**c**) Western blot analysis of lysates from electroporated Caco-2–N cells, which electroporated transcribed RNA transcripts of viral genome. Representative images of SARS-CoV-2 Spike S1 Western blots were shown with GAPDH used as a processing control. (**d**) RT-PCR analysis of generated trVLPs in Caco-2 cells after infection at an MOI of 0.1. RT-PCR analysis was performed via 1% agarose gel electrophoresis. M. Molecular mass markers, marked on the left (Kb). (**e**) Visualization of sequencing results of the viruses harboring a single mutation of nsp16. (**f**) Viral RNA was isolated and purified from Caco-2 cell supernatant 48h after trVLPs (MOI of 0.1) infection. Methyltransferase activity assay was performed *in vitro* using VP39 (n = 3, mean value ± SD). (**g**) Cell lysates were collected 2 hours, 36 hours, and 60 hours post-infection (MOI of 0.1) for quantifying the sgE via qRT-PCR with GAPDH used as a control (n = 3, mean value ± SD). (**h**) The production of sgE from **g** was depicted as AUC. The data were statistically analyzed using unpaired Student’s t test (**f, h**). ns, not significant, **P*$<$0.05, ***P*$<$0.01, ****P*$<$0.001, *****P* < 0.0001.

**
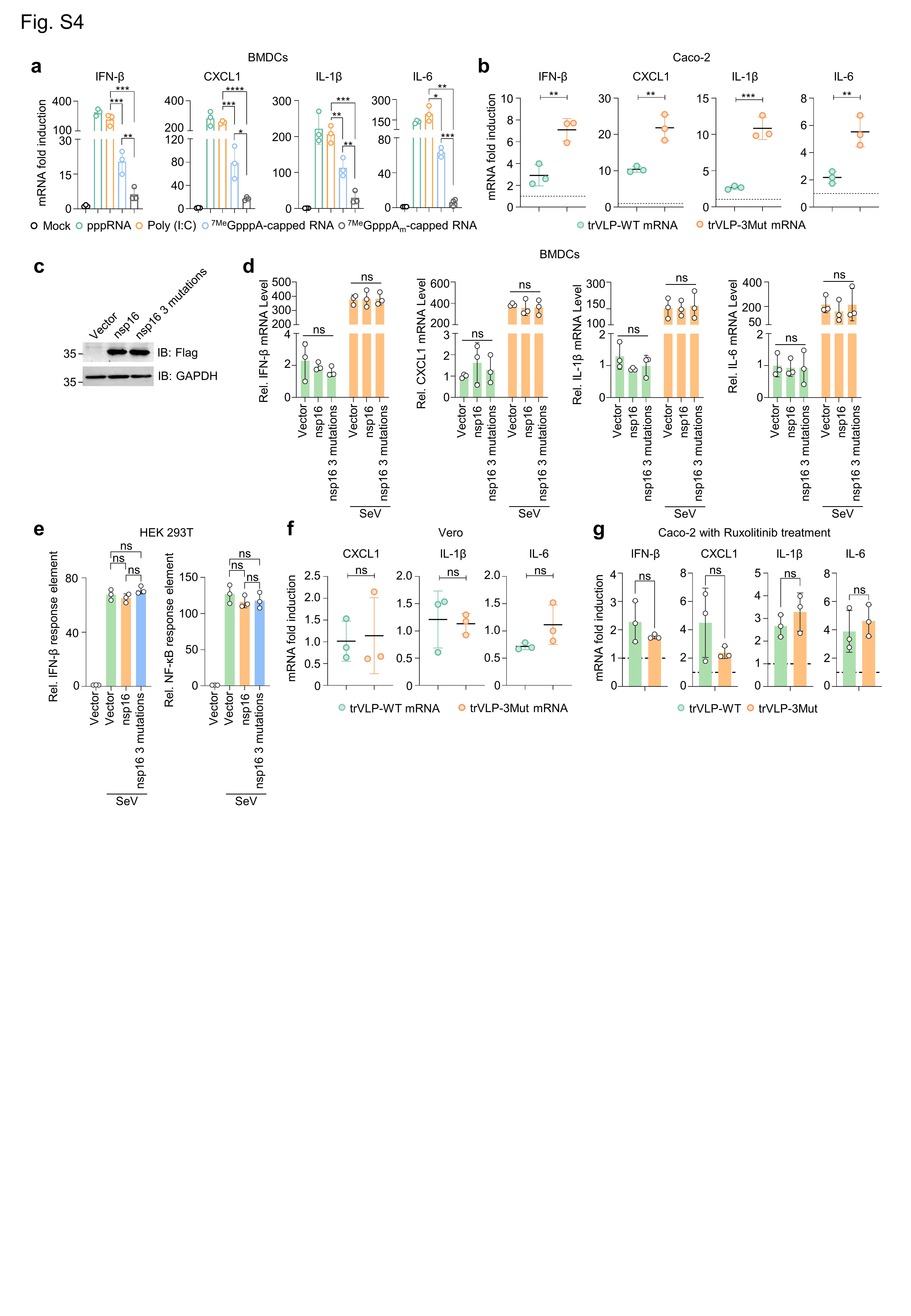
Fig. S4.** **The upregulation inflammatory responses driven by trVLP-3Mut is IFN-I-dependent.** (**a**) BMDCs were transfected with 2 μg of RNA with different 5'-terminal structures (pppRNA, ^7Me^Gppp-capped RNA, and ^7Me^GpppA_m_-capped RNA). mRNA levels of IFN-β and proinflammatory cytokines were analyzed 24 hours post-transfection. Poly (I:C) was used as a positive control (n = 3, mean values ± SD)**.** (**b**) Caco-2 cells were transfected with 2 μg of purified viral mRNA. mRNA expression of IFN-β and proinflammatory cytokines was assessed 24 hours post-transfection (n = 3, mean values ± SD). (**c, d**) BMDCs were transfected with equal amounts of nsp16 and nsp16 3 mutations expression plasmids. Protein expression was examined by Western blot treatment with anti-FLAG and anti-GAPDH antibodies (**c**). mRNA expression of IFN-β and proinflammatory cytokines was detected 12 hours after infection with SeV by qRT-PCR (**d**) (n = 3, mean values ± SD). (**e**) HEK 293T cells were co-transfected with IFN-β-Luc (left), NF-κB-Luc (right), pRL-TK Renilla luciferase reporter plasmid, and vector or nsp16 or nsp16 3 mutations expression plasmids. Luciferase activity was measured 12 hours after infection with SeV (n = 3, mean values ± SD). (**f**) Vero cells were transfected with 2 μg of purified viral mRNA. mRNA expression of IFN-β and proinflammatory cytokines was assessed 24 hours after transfection (n = 3, mean values ± SD). (**g**) 40 μg/ml neutralizing antibody-treated or untreated Caco-2 cells were infected with trVLP-WT or trVLP-3Mut at a MOI of 0.1. mRNA expression of IFN-β and proinflammatory cytokines was assessed 24 hours post-infection (n = 3, mean values ± SD). The dotted lines denoted the RNA copies from the mock (**b, g**). The data were statistically analyzed using unpaired Student’s t test (**a, b, e, f, g**) or two-way ANOVA followed by Turkey’s test (**d**). ns, not significant, **P*$<$0.05, ***P*$<$0.01, ****P*$<$0.001, *****P* < 0.0001.


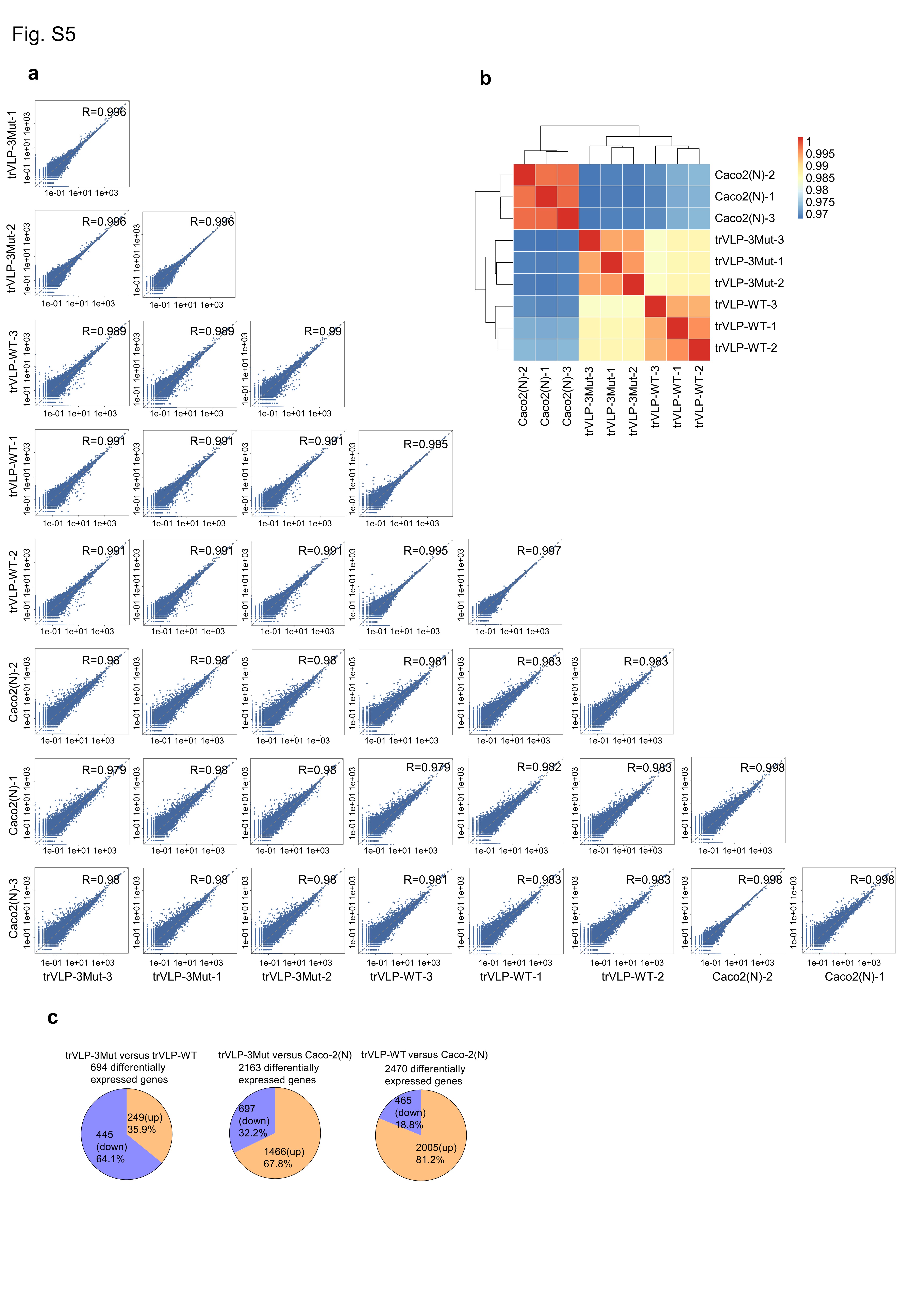


**Fig. S5. Sample correlation analysis and cluster analysis.** (**a**) Pearson correlation coefficients (PCC) between samples used for mRNA expression analysis. The correlations between samples reflect the similarity of gene expression levels; values close to 1 indicate a high degree of similarity between samples. (**b**) Sample clustering analysis was performed based on the Pearson correlation coefficient. (**c**) Pie chart showing the distribution of 694 DEGs between trVLP-WT versus trVLP-3Mut groups, 2163 DEGs between trVLP-3Mut versus control groups and 2470 DEGs between trVLP-WT versus control groups.

**
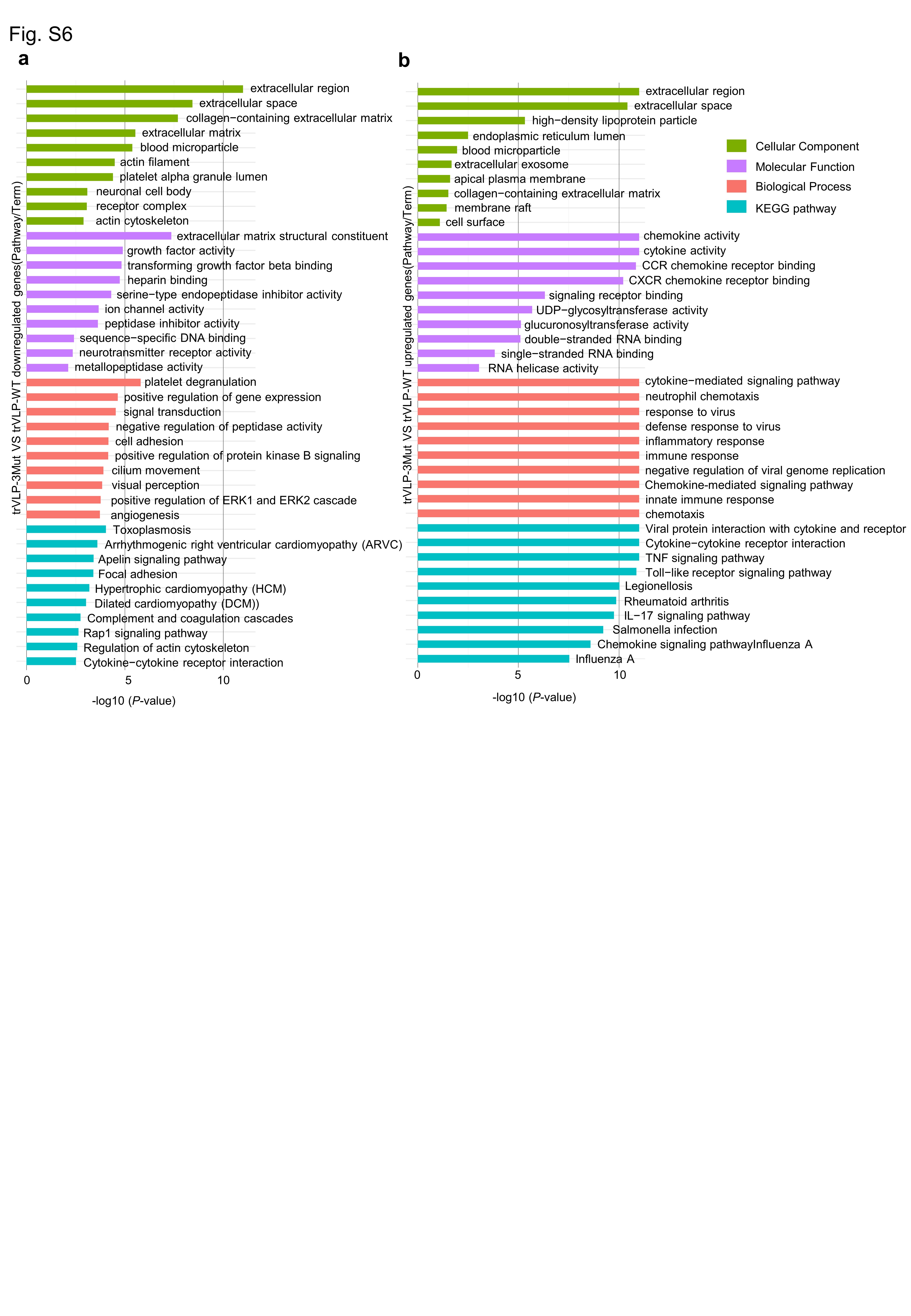
Fig. S6.** **GO-term and KEGG pathway enrichment of DEGs.** (**a and b**) GO-term functional enrichment in three categories (BP, MF, CC) and KEGG pathway analysis were performed for upregulated genes and downregulated genes in Caco-2 cells infected with trVLP-WT or trVLP-3Mut.

**
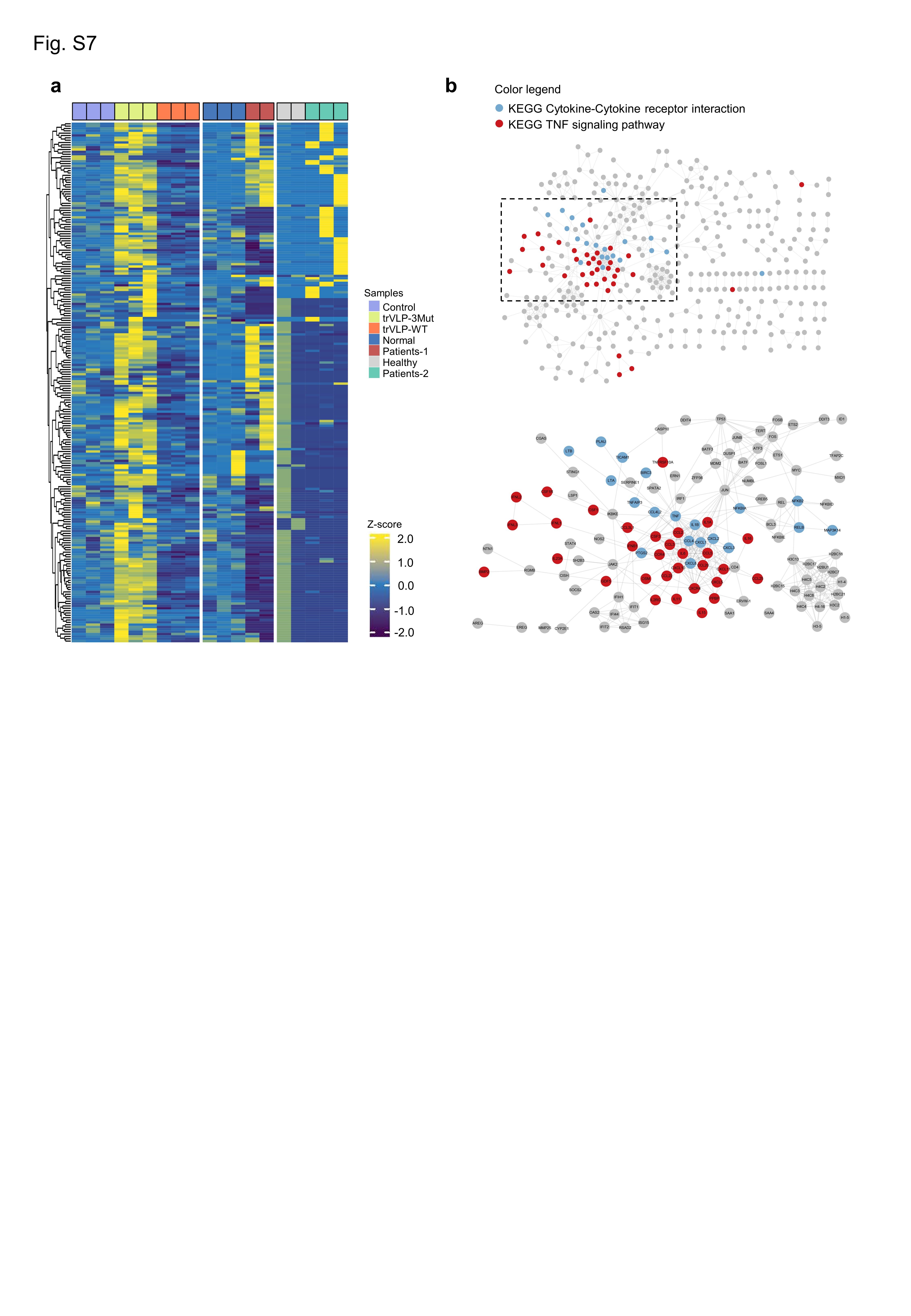
Fig. S7.** **The analysis of transcriptional signature.** (**a**) Heatmap representation of selected shared genes ordered by hierarchical clustering. (**b**) Protein-Protein Interaction (PPI) network of DEGs in trVLP-3Mut comparing to the control. Interactions with confidence scores greater than 0.9 are presented.

**
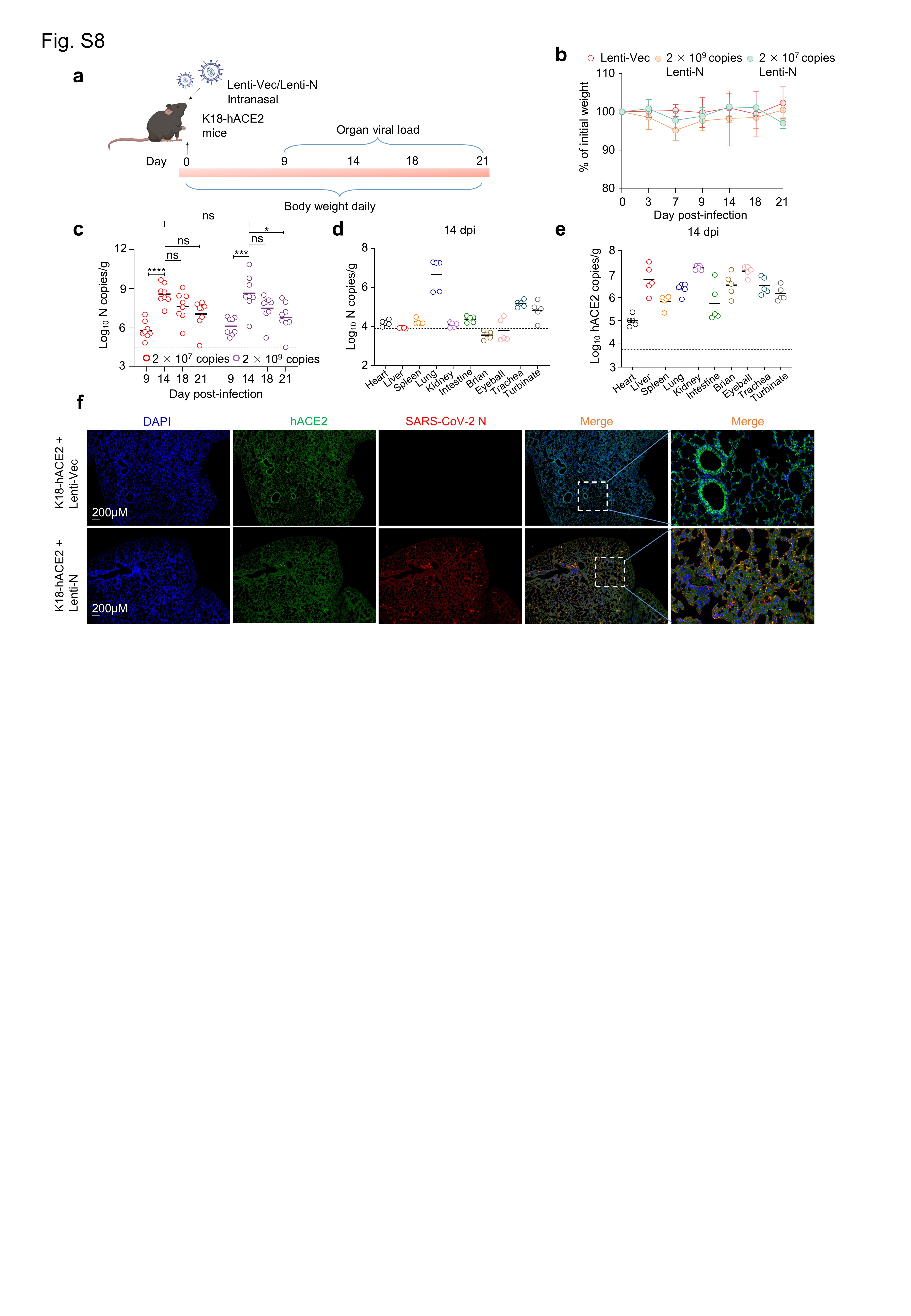
Fig. S8.** **Construction of Lentivirus-N-transduced K18-hACE2 KI mice.** (**a**) Mouse experimental schedule. Six-to eight-week-old male and female K18-hACE2 KI mice were inoculated with Lentivirus-N (Lenti-N, 2 × 10^7^ copies or 2 × 10^9^ copies, intranasal route) or Lentivirus-Vector (Lenti-Vec) for analysis. Images of the mice and viruses were created with BioRender.com. (**b**) Mouse weight change was monitored (n = 5 per group). (**c**) mRNA expression levels of SARS-CoV-2 N in the lung tissues of mice receiving the Lenti-N at 9, 14, 18, 21 dpi. (**d, e**) mRNA expression levels of SARS-CoV-2 N (**d**) and human ACE2 (**e**) in the tissues of mice after Lenti-N transduction at 14 dpi. The dotted lines denoted the limit of detection. (**f**) Immunostaining of lung tissues with a SARS-CoV-2 N protein-specific mAb (red), hACE2 mAb (green) and 4, 6-diamidino-2-phenylindole (DAPI, blue) following Lenti-N transduction at 14 dpi.

**
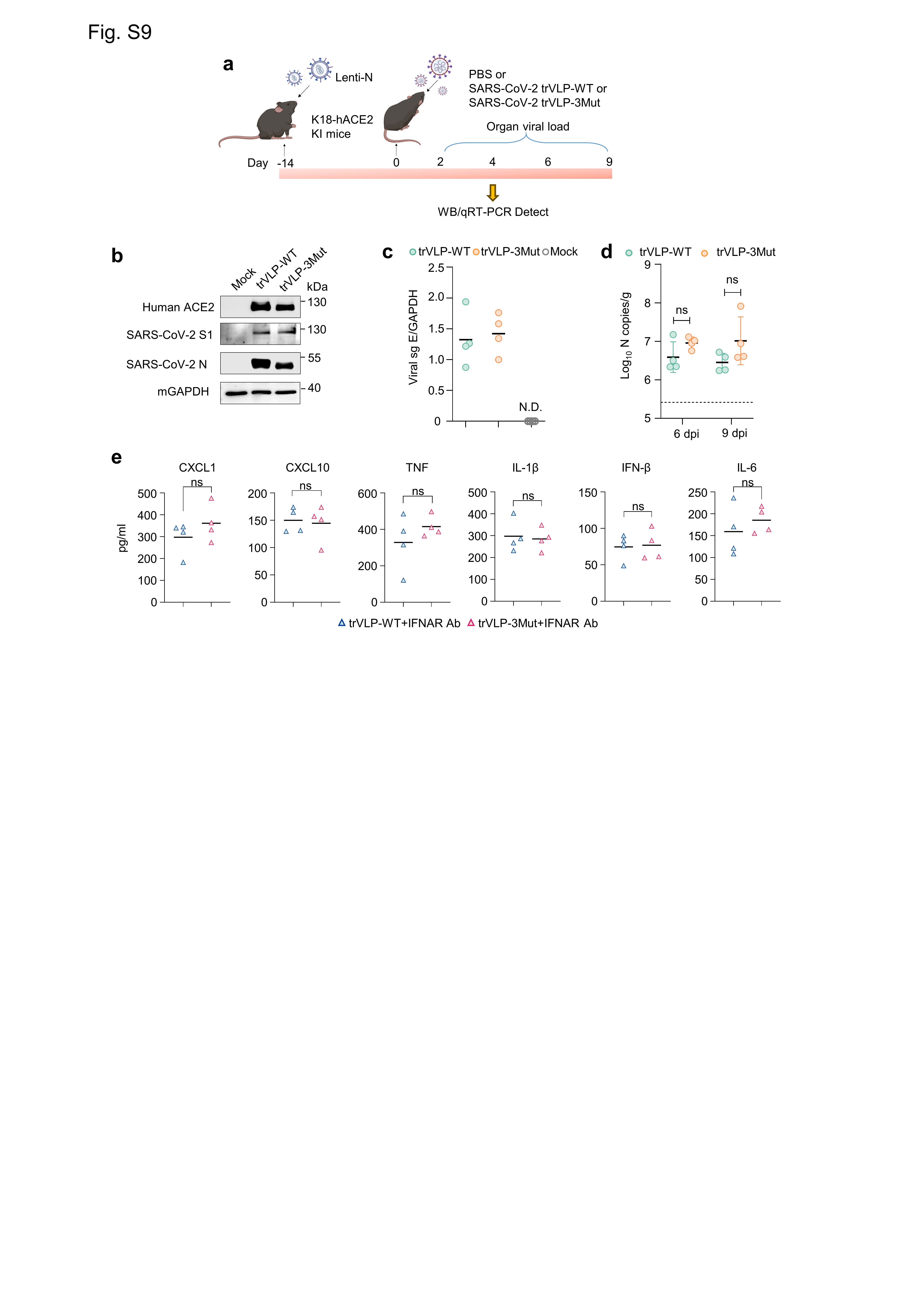
Fig. S9.** **Rescue and validation of trVLPs in K18-hACE2-Lenti-N KI mice.** (**a**) Mouse experimental protocol. Images of the mice and viruses were created with BioRender.com. (**b**) Following infection of trVLPs at 4 dpi, mouse lung tissue lysates were separated by SDS-PAGE, transferred to nitrocellulose membranes, and reacted with anti-Spike S1 antibody, anti-SARS-CoV-2 N antibody, anti-hACE2 antibody, and anti-mouse GAPDH. Representative immunoblotting results were shown. (**c**) Quantification of SARS-CoV-2 sgRNA of trVLP-WT- or trVLP-3Mut-infected mouse lung tissues at 4 dpi by qRT-PCR (n = 4, mean values ±SD). N.D. denoted non-detection. (**d**) mRNA expression of SARS-CoV-2 N in lung tissues of mice were examined after Lenti-N transduction at 6 dpi or 9 dpi (n = 4, mean values ±SD). (**e**) IFN-β and various proinflammatory cytokines in the bronchoalveolar lavage fluid (BALF) of antibody-treated mice were assessed at 6 dpi using ELISA (n = 4 per group). Statistical analysis was performed using two-way ANOVA followed by Tukey’s test (**d**) and unpaired Student’s t test with Welch’s correction (**e**). ns, not significant (*P*$>$0.05), ***P*$<$0.01, ****P*$<$0.001.

**Table S1.** Primers used for transcription in vitro, qRT-PCR and RT-PCR.

| **Primer** | **Sequence (5'→3')** |
| --- | --- |
| SgRNA leader sequence F | CGATCTCTTGTAGATCTGTTCTC |
| SARS-CoV-2 E sgRNA R | ATATTGCAGCAGTACGCACACA |
| Probe-5`6-FAM, 3`BHQ1 | ACACTAGCCATCCTTACTGCGCTTCG |
| hGAPDH Probe-5`CY_5_, 3`BHQ_3_ | CTGCTTAGCACCCCTGGCCA |
| hGAPDH F | CAGCCTCAAGATCATCAGCA |
| hGAPDH R | TGTGGTCATGAGTCCTTCCA |
| mGAPDH F | AGTGTTTCCTCGTCCCGTAG |
| mGAPDH R | CTGTGCCGTTGAATTTGC |
| SARS-CoV-2 E F | ACAGGTACGTTAATAGTTAATAGCGT |
| SARS-CoV-2 E R | ATATTGCAGCAGTACGCACACA |
| SARS-CoV-2 N F | TAATCAGACAAGGAACTGATTA |
| SARS-CoV-2 N R | CGAAGGTGTGACTTCCATG |
| Human ACE2 F | CATTGGAGCAAGTGTTGGATCTT |
| Human ACE2 R | GAGCTAATGCATGCCATTCTCA |
| Human IL-6 F | AGACAGCCACTCACCTCTTCAG |
| Human IL-6 R | TTCTGCCAGTGCCTCTTTGCTG |
| Human IFN-β F | TTCTGCCAGTGCCTCTTTGCTG |
| Human IFN-β R | TGATAGACATTAGCCAGGAG |
| Human IL-8 F | GAGAGTGATTGAGAGTGGACCAC |
| Human IL-8 R | CACAACCCTCTGCACCCAGTTT |
| Human TNF F | CCTCTCTCTAATCAGCCCTCTG |
| Human TNF R | GAGGACCTGGGAGTAGATGAG |
| Human CXCL10 F | GTGGCATTCAAGGAGTACCTC |
| Human CXCL10 R | GCCTTCGATTCTGGATTCAGACA |
| Human CXCL1 F | AGCTTGCCTCAATCCTGCATCC |
| Human CXCL1 R | TCCTTCAGGAACAGCCACCAGT |
| Human IL-1β F | AAGCTGATGGCCCTAAACAG |
| Human IL-1β R | AGGTGCATCGTGCACATAAG |
| RT-PCR F | ACAAGGCCATGGCTGATATCGgatgattttgttgaaataataaaatcccaag |
| RT-PCR WT-3MUT R | GTGGTGGTGGTGGTGGTGCtagttgttaacaagaacatcactagaaataac |
| pppAC_20_ F | TAATACGACTCACTATAACCCCCCCCCCCCCCCCCCCC |
| pppAC_20_ R | GGGGGGGGGGGGGGGGGGGGTTATAGTGAGTCGTATTA |
| pppUC_20_ F | TAATACGACTCACTATATCCCCCCCCCCCCCCCCCCCC |
| pppUC_20_ R | GGGGGGGGGGGGGGGGGGGGATATAGTGAGTCGTATTA |
| pppGC_20_ F | TAATACGACTCACTATAGCCCCCCCCCCCCCCCCCCCC |
| pppGC_20_ R | GGGGGGGGGGGGGGGGGGGGCTATAGTGAGTCGTATTA |
| pppCC_20_ F | TAATACGACTCACTATACCCCCCCCCCCCCCCCCCCCC |
| pppGC_20_ R | GGGGGGGGGGGGGGGGGGGGGTATAGTGAGTCGTATTA |
| SARS-COV-2 5’UTR F | TAATACGACTCACTATAAGGAAAGGTTTATACCTTCCCAGGTAACAA |
| SARS-CoV-2 5’UTR R | CTTACCTTTCGGTCACACCCG |
| SARS-CoV 5’UTR F | TAATACGACTCACTATAAGATTAGGTTTTTACCTACCCAGGAAAAGC |
| SARS-CoV 5’UTR R | CTTACCTTTCGGTCACACCCGGAC |
| MERS-CoV 5’UTR F | TAATACGACTCACTATAGATTTAAGTGAATAGCTTGG |
| MERS-CoV 5’UTR F | GATGTGCCCCGAATTGCCACG |
| MHV-CoV 5’UTR F | TAATACGACTCACTATAGTATAAGAGT GATTGGCGTC |
| MHV-CoV 5’UTR R | TATGCAACCTATGGGTGGGC |

F, Forward primer. R, Reverse primer. nsp, indicates non-structural protein.
